# Bupropion decreases plasma levels of asymmetric dimethylarginine and ameliorates renal injury by modulation of Ddah1, Oatp4c1, Oct2, and Mate1 in rats with adenine-induced chronic renal injury

**DOI:** 10.3389/fphar.2025.1565713

**Published:** 2025-05-22

**Authors:** Lulu Huang, Yun Xiao, Xiaoyu Han, Yang Yu, Chao Zheng, Xiangdong Fang, Qing Li, Fanglan Liu, Chunhua Xia, Yongjie Zhang, Jiake He

**Affiliations:** ^1^ Department of Pharmacy, The 2nd affiliated hospital, Jiangxi Medical College, Nanchang University, Nanchang, Jiangxi, China; ^2^ Clinical Pharmacology Institute, School of Pharmacy, Jiangxi Medical College, Nanchang University, Nanchang, Jiangxi, China; ^3^ School of Pharmacy, Jiangxi Medical College, Nanchang University, Nanchang, Jiangxi, China; ^4^ Department of Nephrology, The 2nd affiliated hospital, Jiangxi Medical College, Nanchang University, Nanchang, Jiangxi, China; ^5^ Department of Pathology, The 2nd affiliated hospital, Jiangxi Medical College, Nanchang University, Nanchang, Jiangxi, China; ^6^ Clinical Pharmacokinetics Laboratory, School of Basic Medicine and Clinical Pharmacy, China Pharmaceutical University, Nanjing, China

**Keywords:** bupropion and its metabolites, asymmetrical dimethylarginine, chronic renal injury, renal transporters, metabolism enzyme

## Abstract

**Objective:**

The objective of the study was to investigate whether bupropion (BUP) or its circulation metabolites could decrease plasma level of asymmetric dimethylarginine (ADMA) and ameliorate renal injury by modulation of Ddah1, Oatp4c1, Oct2, and Mate1 in rats with adenine-induced chronic renal injury.

**Methods:**

The study initially determined the effect of BUP and its metabolites on cell viability and apoptosis in HK2 cells in the presence and absence of ADMA. Secondly, the study explored whether long-term administration of BUP could reduce the plasma level of ADMA and mitigate renal damage. Thirdly, the expression and activity of Oct2, Ddah1, Mate1 and Oatp4c1 was determined by Western blot and UPLC-MS/MS.

**Results:**

With 0.5 μmol/L ADMA, hydroxybupropion (HBUP, 100 nmol/L), threo-hydrobupropion (TBUP, 10 nmol/L and 1 μmol/L) reduced N-Acetyl-β-D-glucosidase (NAG) level. At 5 μmol/L ADMA, BUP (1 nmol/L-1 μmol/L), HBUP (1–100 nmol/L), and BUP cocktail enhanced survival. At 50 μmol/L ADMA, HBUP (10 nmol/L and 1 μmol/L), TBUP/erythro-hydrobupropion (EBUP) (10–100 nmol/L), and BUP cocktail stimulated survival. EBUP (1 and 100 nmol/L) lowered LDH. BUP (100 nmol/L) and TBUP (1 μmol/L) decreased NAG. TBUP (10 nmol/L, 1 μmol/L) and EBUP (100 nmol/L) inhibited apoptosis. In adenine-induced chronic renal injury rats, long-term administration of BUP significantly decreased the serum concentration of ADMA and creatinine by 12.78% and 38.85%, respectively, ameliorated interstitial lesions and fibrosis and upregulated Ddah1, Oatp4c1, Oct2, Mate1. BUP increased metformin renal clearance without affecting digoxin disposition.

**Conclusion:**

Bupropion moderately decreases plasma levels of ADMA and ameliorates renal injury by modulation of Ddah1, Oatp4c1, Oct2, and Mate1.

## Introduction

Chronic kidney disease (CKD), with an estimated prevalence of approximately 10.0% (95% confidence interval 8.5–11.4) worldwide, has emerged as a global public health challenge (Sundström et al., 2022; Guo et al., 2025). Epidemiological studies in China indicated that the prevalence of CKD among adults is 8.2% (Wang et al., 2023). The accumulation of various endogenous and exogenous compounds, particularly uremic toxins, trigger multiple comorbidities as CKD progresses (Lim et al., 2021). Moreover, the prevalence of depression among CKD patients adversely affects the quality of life and significantly increases the duration of hospitalization and mortality (Charles and Ferris, 2020; Naber and Purohit, 2021; Nagler et al., 2012).

Uremic toxins, lead to various degrees of cellular, tissue, and organ damage (Brunini et al., 2006; Chen and Chiang, 2021). They may also cause immune dysregulation, inflammatory injury, and metabolic disturbances (Brunini et al., 2006; Chen and Chiang, 2021). Asymmetric dimethylarginine (ADMA) is an amine toxin among small molecular uremic toxins and serves as an endogenous inhibitor of nitric oxide synthase. It induces oxidative stress responses and is involved in endothelial dysfunction, as well as the onset and progression of cardiovascular diseases in patients with CKD (Oliva-Damaso et al., 2019; Shafi et al., 2017; Strobel et al., 2013). Serum levels of ADMA are important biomarkers for CKD and are negatively correlated with residual renal function. In patients with end-stage renal disease, serum concentrations of ADMA increased by 4–10 times (Oliva-Damaso et al., 2019; Shafi et al., 2017).

The kidneys play a crucial role in the systematic elimination of ADMA. More than 80% ADMA is degraded by dimethylarginine dimethylaminohydrolase 1 (Ddah1) into citrulline and dimethylamine in proximal tubular epithelial cells, while symmetric dimethylarginine, the isomer of ADMA, is mainly excreted unchanged in the urine (Oliva-Damaso et al., 2019; Tain and Hsu, 2017; Guo et al., 2023). Strobel J et al. reported that ADMA is a substrate for both the basolateral organic cation transporter 2 (Oct2) and the apical multidrug and toxin extrusion protein 1 (Mate1) in renal tubular epithelial cells (STROBEL et al., 2013). Organic anion-transporting polypeptide 4c1 (Oatp4c1), the only Oatps expressed in the kidney, also involves ADMA renal excretion (Taghikhani et al., 2019; Yamaguchi et al., 2010; Mikkaichi et al., 2004; Toyohara et al., 2009). Rat Oatp4c1 traffics to the apical cell surface of polarized epithelium and localizes primarily in the proximal straight tubules, the S3 fraction of the nephron, facilitating substrate reabsorption (Toyohara et al., 2009; He et al., 2014). Both acute and chronic kidney failure alters the expression of Oct2, Mate1, and Oatp4c1, causing reduced renal clearance (CL_renal_) of protein-bound toxins (Masereeuw et al., 2014; Schwenk and Pai, 2016; Naud et al., 2011). Toyohara T et al. found that modulation of Oatp4c1 expression and activity not only directly correlates with the CL_renal_ of protein-bound toxins, such as ADMA, transuranic acid, and guanidinosuccinic acid, but also improves kidney dysfunction related hypertension, myocardial hypertrophy and renal inflammatory responses (Toyohara et al., 2009).

Bupropion (BUP), a dopamine-norepinephrine reuptake inhibitor, is prescribed to treat major depressive disorder and to aid smoking cessation or obesity management (Greig and Keating, 2015; Cinciripini et al., 2017; Jefferson et al., 2005). It exhibits high safety profiles, with no significant cardiac or renal toxicity (Zyban, 2014). BUP undergoes extensive metabolism via Cytochrome P450 (CYP) 2B6 and carbonyl reductase to form active metabolites hydroxybupropion (HBUP), erythro-hydrobupropion (EBUP), and threo-hydrobupropion (TBUP) (Jefferson et al., 2005; Connarn et al., 2015). This process is followed by uridine diphosphate glycosyltransferase (UGT) 2B7 and UGT1A9 mediated stereoselective glucuronidation that produces various inactive glucuronide metabolites for excretion (Sager et al., 2016; Gufford et al., 2016). The fraction of unchanged BUP excreted in urine accounts for only 0.5%, while less than 1% is eliminated through feces (Khan et al., 2016). After long-term administration of BUP, the steady-state peak plasma concentration (C_max_) and the area under the plasma concentration time curve (AUC) for HBUP and TBUP were 4-, 7-fold, and 4-, 6-fold higher than those following single-dose of BUP, respectively (Costa et al., 2019; Benowitz et al., 2013). Mao et al. demonstrated that neither BUP nor its active metabolites were substrates for Oct1, Oatp1b1, Oatp1b3, Oatp2b1, Oatp4a1, breast cancer resistance protein (Bcrp), multidrug resistance-associated protein 2 (Mrp2) or P-glycoprotein (P-gp) (Han et al., 2019).

Our precious studies reported that at clinically relevant plasma concentrations, BUP and its metabolites activated human-OATP4C1 mediated digoxin (DIG) tubular secretion and inhibited rat-Oatp4c1-mediated DIG renal reabsorption (He et al., 2014). We also found that multiple-dosing of BUP significantly increased oral DIG CL_renal 0–48h_ and CL_non-renal0–48h_ in cynomolgus monkeys, suggesting the capability of transporter and enzyme modulation (Shen et al., 2018). Therefore, it is hypothesized that at clinically relevant plasma concentrations, BUP or its metabolites, modulated Oct2, Oatp4c1, and Mate1 mediated renal transport of ADMA and/or Ddah1 mediated metabolism, which could alleviate the exacerbation of ADMA and retard the progression of renal interstitial lesions and fibrosis.

To test this hypothesis, the study initially determined the effect of BUP and its metabolites on cell viability and apoptosis in HK2 cells both in the presence and absence of ADMA. Secondly, the study explored whether the long-term administration of BUP could reduce the plasma level of harmful uremic toxins such as ADMA and mitigate renal damage through histopathological examination in the chronic renal injury model in rats. Thirdly, the chronic effect of BUP and its metabolites on the expression of Oct2, Ddah1, Mate1 and Ddah1 was determined. The activity of the transporters, as measured by the pharmacokinetics of the probe drugs, was also evaluated using UPLC-MS/MS.

## Materials and methods

### Chemicals and reagents

BUP (purity >99.9%), HBUP (purity >99.9%), TBUP (purity >99.9%), EBUP (purity >99.9%), ADMA (purity >99.9%), metformin (MET, purity >99.9%) and furosemide (FUR, purity >99.9%) were purchased from National Standard Pharmaceutical Group Co., Ltd. (Beijing, China). Digoxin (DIG, purity >99.9%) were purchased from J&K Scientific Ltd. (Shanghai, China). Adenine (purity >99.5%) was purchased from Shanghai Aladdin Biochemical Technology Co., Ltd. (Shanghai, China). Rosuvastatin (RSV, purity >95.0%) was purchased from J&K Scientific Co., Ltd. (Shanghai, China). Diazepam (DIA, purity >99.0%) was purchased from Sigma Aldrich (St Louis, MO, United States). DIG injection (0.25 mg/mL) was purchased from Southwest Pharmaceutical Co., Ltd. (Chongqing, China). FUR injection (10 mg/mL) was purchased from Henan Runhong Pharmaceutical Co., Ltd. (Chongqing, China). Cell count kit-8 (CCK-8) was purchased from GLP Bio Co., Ltd. (Montclair, United States). Acridine Orange (AO)/ Ethidium Bromide (EB) double fluorescence staining kit was purchased from Phygene Biotechnology Co., Ltd. (Fuzhou China). Lactate dehydrogenase (LDH) assay kit, creatinine (Cre) assay kit, urea nitrogen (Bun) assay kit and albumin (Alb) assay kit were purchased from Nanjing Jiancheng Bioengineering Institute (Nanjing, China). N-Acetyl-β-D-glucosidase (NAG) activity assay kit was purchased from Solarbio Co., Ltd. (Beijing, China). ADMA elisa assay kit, kidney injury molecule-1 (Kim-1) elisa assay kit, cystatin-c (Cys-c) elisa assay kit, β2-MG elisa assay kit were purchased from Shanghai Jingkang Biotechnology Co., Ltd. (Shanghai, China). Antibodies against Oct2 (13594-1-AP), Mate1 (20898-1-AP), Oatp4c1 (24584-1-AP), and β-actin (20536-1-AP) were purchased from Wuhan Protech Biotechnology Co., Ltd. (Wuhan, China). Antibodies against Ddah1 (PB1052) was purchased from Boster Biotechnology Co., Ltd. (Wuhan, China).

### Ethical approval

All experimental procedures were performed in accordance with the Institutional Guidelines for the Care and Use of Laboratory Animals and were approved by the Institutional Animal Care and Use Committee of Nanchang University in 2022 (Permit Number: NCULAE-20221031034). Humane end point:I. rats lost more than 20% of their normal animal body weight. II. rats were unable to take food or water. III. abnormal manifestations such as jaundice, excessive depression and self-harm in rats. In cases of hepatotoxicity, rats may exhibit symptoms such as jaundice, lethargy, reduced activity, weight loss and ruffled fur.

### Cell culture

Human renal cortical proximal tubular epithelial cell (HK-2) was obtained from Procell Life Science & Technology Co., Ltd. (Wuhan, China). Cells were cultured in DMEM-F12 medium supplemented with 10% fetal bovine serum, 100 U/mL penicillin and 100 μg/mL streptomycin in the ratio of 100:10:1:1 at 37 °C in the presence of 5% CO_2_.

### Cell counting Kit‐8 (CCK8) assay

Cell viability was assessed by CCK8 assay according to the manufacturer’s protocols. Briefly, cells were seeded and cultured in 96-well plates at a density of 5 × 10^3^/ well in 100 μL of medium. Then the cells were treated with varying concentrations of ADMA, BUP or its metabolites or BUP cocktail, and then incubated for 24 h at 37 °C. Then CCK‐8 reagent was added to each well and then cultured for 2 h at 37 °C. The absorbance at 450 nm was measured using a multilabel reader (PerkinElmer EnSpire, United States). All experiments were performed in triplicate.

### Acridine Orange/Ethidium Bromide (AO/EB) staining

Cell apoptosis was assessed by AO/EB dual staining according to the manufacturer’s protocol. Briefly, cells were grown in 96-well plates at a density of 5 × 10^3^/ well in 100 μL of medium. Then the cells were treated with varying concentrations of ADMA, BUP, BUP metabolites or BUP cocktail, and then incubated for 24 h at 37 °C. Then a solution containing 2.5 μL AO and 2.5 μL EB was added to each well and then incubated for 5 min at 37 °C. Cell fluorescence was measured using an inverted fluorescence microscope (Olympus, Japan).

### Biochemical examination in the culture medium and cell lysate

LDH activity in the culture medium and NAG activity in cell lysate was determined using LDH kit and NAG kit according to the manufacturer’s instructions, respectively.

### Animals

Male Sprague-Dawley (SD) rats (200–220 g) were obtained from Hunan SJA Laboratory Animal Co., Ltd. (Changsha, China, No. SCXK<Xiang> 2019–0004). All 30 rats were kept at 12 h light and 12 h dark cycle with 50%–60% humidity at 25°C ± 2°C and given standard sterile food and water. Standard sterile food consists of crude protein (≥180 g/kg), crude fat (≥40 g/kg), crude fiber (≤50 g/kg), crude ash (≤80 g/kg), moisture and other volatile matter (≤100 g/kg), calcium (10–18 g/kg), total phosphorus (6–12 g/kg) as well as amino acids, vitamins and minerals. They were randomly assigned to respective experiment groups. Whenever overnight fasting was used before dosing, food was provided 2 h after dosing. All animal care and experimental procedures were conducted in accordance with the guidelines for the care and use of laboratory animals issued by NIH, and the animal experimental protocol was approved by the Ethics Committee of Nanchang University (NCULAE-20221031034).

### Establishment of a chronic renal injury model in rats

Following 1 week of acclimatization, venous blood was collected to measure baseline plasma levels of creatinine (Cre) and blood urea nitrogen (Bun). A daily gavage of adenine suspension (200 mg/kg) was administered for a duration of 21 days (Yang et al., 2024). On day 22, venous blood samples were taken to assess Cre and Bun levels. Pathological observation of renal tissues was also conducted for model validation.

### Animal study protocol

For the plasma pharmacokinetic study, 30 rats with chronic renal injury were randomly assigned into six groups (n = 5, each group, groups I to VI) and received oral gavage (p.o) of BUP (40 mg/kg) or an equivalent volume of physiological saline as vehicle control for 27 days. On day 28, rats in group I were treated with a p.o. dose of vehicle control. Rats in group II were treated with a p.o. dose of BUP at 40 mg/kg. Rats in group Ⅲ were treated with a p.o. dose of vehicle control and a single intravenous (i.v.) dose of DIG at 0.005 mg/kg. Rats in group Ⅳ were treated with a p.o. dose of BUP at 40 mg/kg of and a single i.v. dose of DIG at 0.005 mg/kg. Rats in group V were treated with a p.o. dose of vehicle control and a probe drug cocktail (a single i.v. dose of MET at 5 mg/kg, a single i.v. dose of FUR at 4 mg/kg and a single p.o. dose of RSV at 25 mg/kg). Rats in group VI were treated with a p.o. dose of BUP at 40 mg/kg of and the same probe drug cocktail Blood samples were collected at 0, 5, 10, 30, 60, 120, 180, 360, 480 and 720 min in group I-II, and at 0, 2, 5, 15, 30, 60, 120, 240, 360, 480, 600, and 720 min in group III-VI (0.1 mL each for the first two time points and 0.2 mL each for the remaining time points). After each sampling, a volume of 0.2 mL physiologic saline solutions was administered to sustain isotonic fluid balance. Blood samples were centrifuged for plasma collection at 3,000 rpm for 10 min at 4°C. Urine samples were collected at 0–6 h and 6–12 h though metabolic cages. All samples were stored at −80°C until analysis by reagent kit and UPLC-MS/MS.

### Biochemical examination and histopathology in rats

After the last sample in the plasma pharmacokinetic study, all rats were deeply anesthetized using an anesthetic agent, 50 mg/kg of sodium pentobarbital (ip), as a chemical method of euthanasia, and exsanguinated by severing the abdominal aorta. Blood and kidney samples were harvested. The plasma samples were used for the assay of ADMA, Kim-1, Cys-C, β2-MG, Cre, Bun and Alb according to the manufacturer’s instructions, respectively. The urine samples were used for the assay of ADMA, Kim-1, β2-MG and Cre according to the manufacturer’s instructions, respectively. Kidney specimens were fixed in 4% paraformaldehyde for 2–3 days and passed through the routine paraffin embedding procedures. They were serially sectioned and stained with hematoxylin and eosin, masson, and sirius red for histological evaluations.

### Western blot analysis

Total proteins were extracted from kidney tissues using radio immunoprecipitation assay lysis (RIPA) buffer supplemented with phenylmethanesulfonyl fluoride (PMSF). Samples were then separated on 10% SDS-polyacrylamide gels and subsequently transferred to polyvinylidene difluoride (PVDF) membranes via electroblotting. The membranes were blocked with 5% skim milk powder for 2 h and incubated overnight at 4 °C with the appropriate primary antibody, followed by a 1 h incubation at room temperature with a secondary antibody conjugated to horseradish peroxidase (HRP). The reaction products were visualized through chemiluminescence detection utilizing the enhanced chemiluminescence (ECL) Protein Blotting Detection System. Quantification of the results was performed using ImageJ software.

### Plasma and urine analysis

Biological sample preparation and analysis were performed according to our previous established method with minor modification (He et al., 2014; Shen et al., 2018; Huang et al., 2025). A Shimadzu Nexera liquid chromatography system (Shimadzu, Kyoto, Japan) and an 8050 CL triple quadruple tandem mass spectrometer (Shimadzu, Kyoto, Japan) coupled with an electro-spray ionization (ESI) source was used for the LC-MS/MS analysis of the plasma and urine samples.

Chromatographic separation was conducted at 40 °C with a gradient mobile phase programme to separate BUP, HBUP, EBUP, TBUP, DIG and diazepam (DIA, internal standard) on a Shimadzu Shim-pack Gist-C18 2.0 μm (2.1 × 100 mm; Shimadzu, Japan) analytical column. The mobile phase consisted of water (A), containing 0.1% formic acid containing 5 mM ammonium formate, and methanol (B). The flow rate was 0.4 mL/min and the mobile phase was run using the following gradient programme (B concentration in parentheses): 0.02–2 min (37%–40%), 2.0–6.0 min (40%–65%), 6.0–7.0 min (65%–95%), 7.0–8.0 min (95%), 8.0–8.01 min (95%–40%) and 8.02–10.0 min (37%). The injection volume was 3 μL.

Chromatographic separation was conducted at 30 °C with a gradient mobile phase programme to separate MET, RSV, FUR and DIA (internal standard) on a Shimadzu Shim-pack Gist-C18 3.0 μm (2.1 × 100 mm; Shimadzu, Japan) analytical column. The flow rate was 0.4 mL/min and the mobile phase was run using the following gradient programme (B concentration in parentheses): 0.8 min (1%), 0.8–0.9 min (1%–35%), 0.9–6.0 min (35%–60%), 6.0–6.5 min (60%–95%), 6.5–7.5 min (95%), 7.5–7.6 min (95%–1%) and 7.6–9.0 min (1%). The injection volume was 5 μL.

The following multiple reaction monitoring (MRM) transitions were used: BUP, m/z 239.9→166.05; HBUP, m/z 256.1→167.1; EBUP/TBUP, m/z 242.0→168.2; DIG, m/z 825.4→649.35; MET, 130.20 → 60.20; RSV, 482.00→258.15; FUR, 329.00→205.05; DIA, m/z 285.1→154.1.

### Pharmacokinetic analysis

Phoenix^®^ WinNonlin^®^ version 8.1 software (Pharsight Corporation, Mountain View, CA) was used to calculate the pharmacokinetic parameters in rats by the non-compartmental method. The C_max_ and time to reach C_max_ (T_max_) were obtained directly from the plasma concentration time profile. The AUC was estimated by the trapezoidal rule. CL_renal_ was calculated as the excreted amount in 12 h urine collections divided by the plasma AUC_0–12h_ (Shen et al., 2018). Creatinine clearance (CL_cr_) was calculated from the following formula: CL_cr_ = Urine_cr_ × V/Plasma_cr_ and expressed in mL/h, where Urine_cr_ = urine concentration of Cr; V = urine flow rate (mL/h); Plasma_cr_ = plasma concentration of Cr (Shen et al., 2018). Renal clearance of ADMA (CL_renal_ _ADMA_) was calculated accordingly.

### Statistical analysis

Statistical analyses were performed using GraphPad Prism 8.0 (GraphPad Software, San Diego, CA, United States). The results are presented as mean ± standard deviation (S.D.), unless otherwise stated. As for sample size justification, by assuming comparing the renal clearance of two groups, α = 0. 05, β = 0.10, *Z* = 1.96, *σ* = 4.4979 (He et al., 2014), *E* = 4, *n*1 = *n*2 = *Z* ^2^×*σ* ^2^​/*E* ^2^ = 5 for each group in the *in vivo* experiments. Taking together the 3R principle (reduction, refinement and replacement) and animal welfare of experimental animals, five animals were selected. Additionally, by assuming comparing two groups of cell viability, α = 0. 05, β = 0.10, *Z* = 1.96, *σ* = 35.3479 (obtained from previous pilot study), *E* = 40, *n*1 = *n*2 = = *Z* ^2^×*σ* ^2^​/*E* ^2^ = 3 for each group in the *in vitro* experiments. In addition, the same sample size was adopted for *in-vitro* and in-vivo study in our previous study (He et al., 2014).

Pharmacokinetic parameters, such as area under the plasma concentration-time curve (AUC), C_max_ and clearance (CL), were log-transformed to fit a normal distribution. After testing for homogeneity of variance, statistical analysis was performed using one-way ANOVA followed by LSD *post hoc* test or Dunnett’s T3 *post hoc* test. Nonparametric statistics (Kruskal–Wallis one-way analysis) were used if the data were not normally distributed, such as terminal half-life (T_1/2_) and T_max_. A probability (p) of less than 0.05 was statistically significant.

## Results

### Cytotoxicity in HK2 cells

**FIGURE 1:**
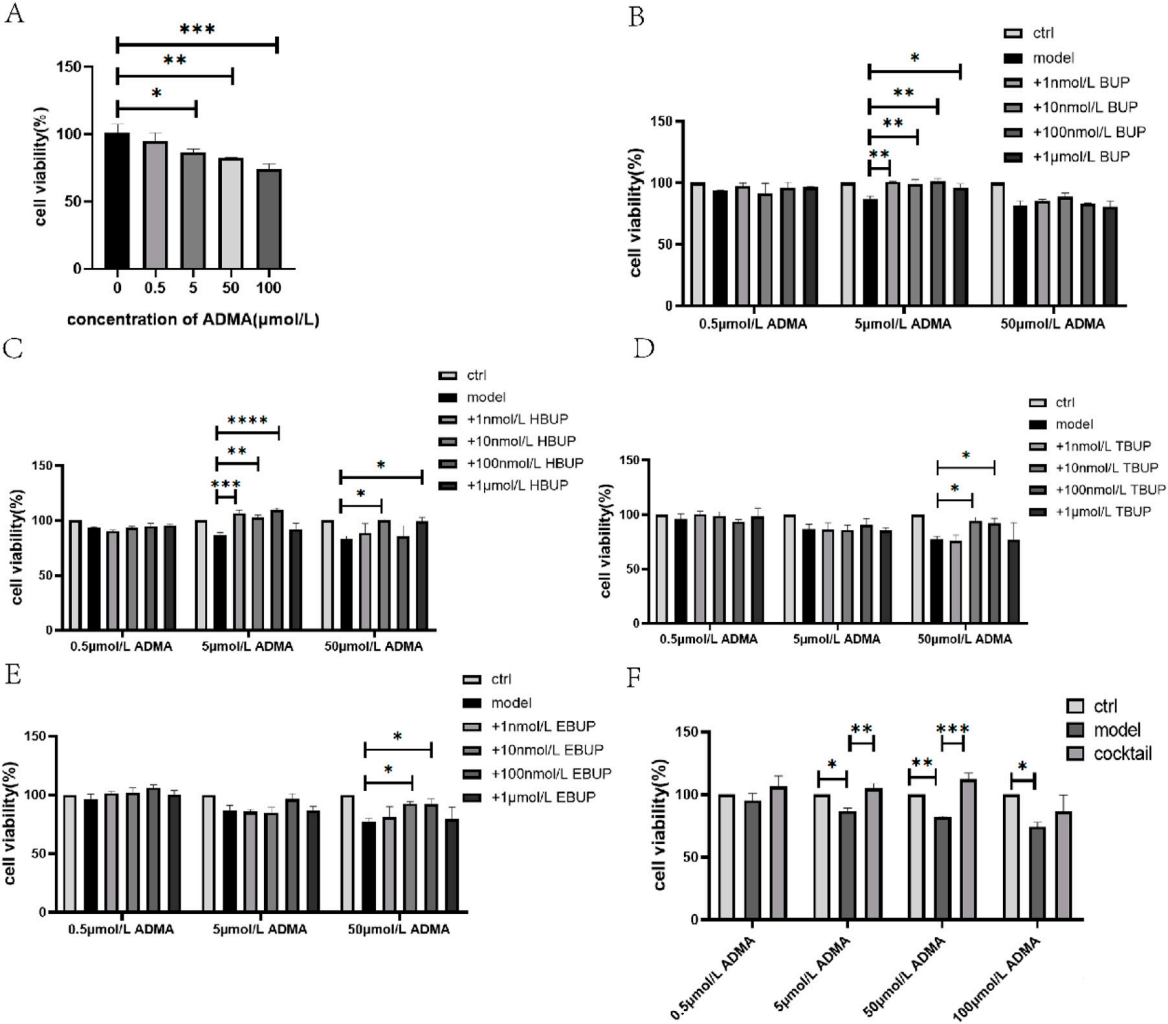
HK2 cell survival in the presence of ADMA **(A)**, BUP **(B)**, HBUP **(C)**, TBUP **(D)**, EBUP **(E)** or BUP cocktail **(F)**. Ctrl means normal cell group. BUP cocktail consisted of 15 nmol/L BUP+125 nmol/L HBUP+50 nmol/L EBUP+50 nmol/L TBUP. Data are expressed as mean ± SD (n = 3). *P < 0.05, **P < 0.01, ***P < 0.001, ****P < 0.0001. Data were analyzed by one-way ANOVA.

**FIGURE 2:**
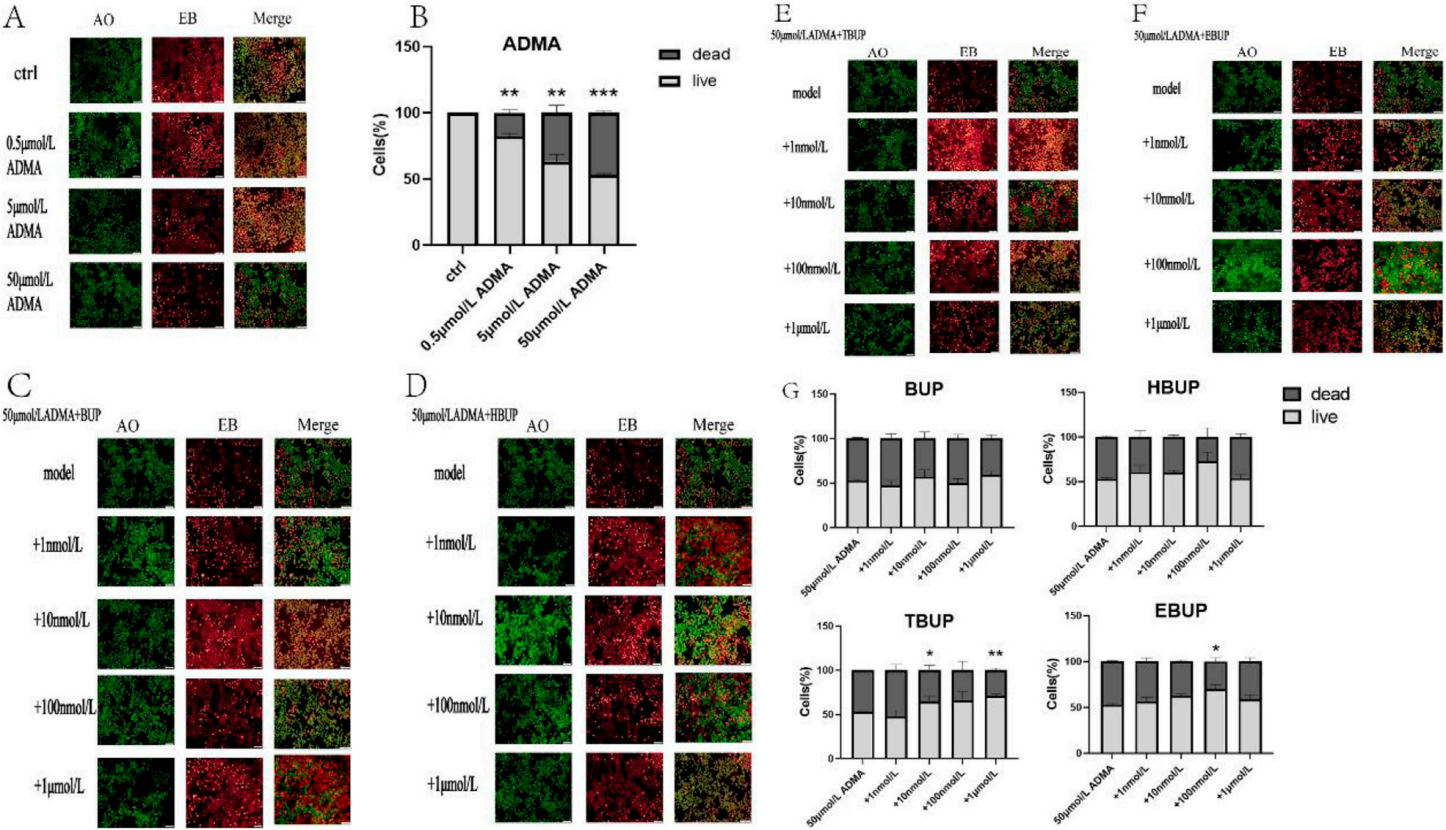
Cell apoptosis with AO/EB staining. **(A,B)** different concentrations of ADMA; **(C–G)** 50 μmol/L ADMA + BUP/HBUP/TBUP/EBUP. Ctrl means normal cell group. *: compared with the first column of each panel. Data are expressed as mean ± SD (n = 3). *P < 0.05, **P < 0.01, ***P < 0.001. Data were analyzed by one-way ANOVA.

Dose-dependent cytotoxicity of ADMA was observed in HK2 cells after 24 h incubation (Figures 1A, 2A).

In the presence of 0.5 μmol/L ADMA, BUP, BUP metabolites and BUP cocktail did not affect cell proliferation during incubation (Figures 1B–F).

In the presence of 5 μmol/L ADMA, BUP (1 nmol/L-1 μmol/L) and its major metabolite, HBUP (1 nmol/L-100 nmol/L), significantly stimulated cell survival. BUP cocktail also significantly stimulated cell survival by 20.96% (Figures 1B–F).

Moreover, in the presence of 50 μmol/L ADMA, HBUP at 10 nmol/L and 1 μmol/L, TBUP at 10 nmol/L and100 nmol/L, EBUP at 10 nmol/L and100 nmol/L and BUP cocktail significantly stimulated cell survival by 17.32%, 16.09%, 17.10%, 14.96%, 15.51%, 15.33%, and 36.33%, respectively (Figures 1B–F). Additionally, the administration of 10 nmol/L and 1 μmol/L TBUP, as well as 100 nmol/L EBUP, effectively mitigated cellular apoptosis (Figure 2G).

### The effect of BUP and its metabolites on the biomarkers in the culture medium and cell lysate

**FIGURE 3:**
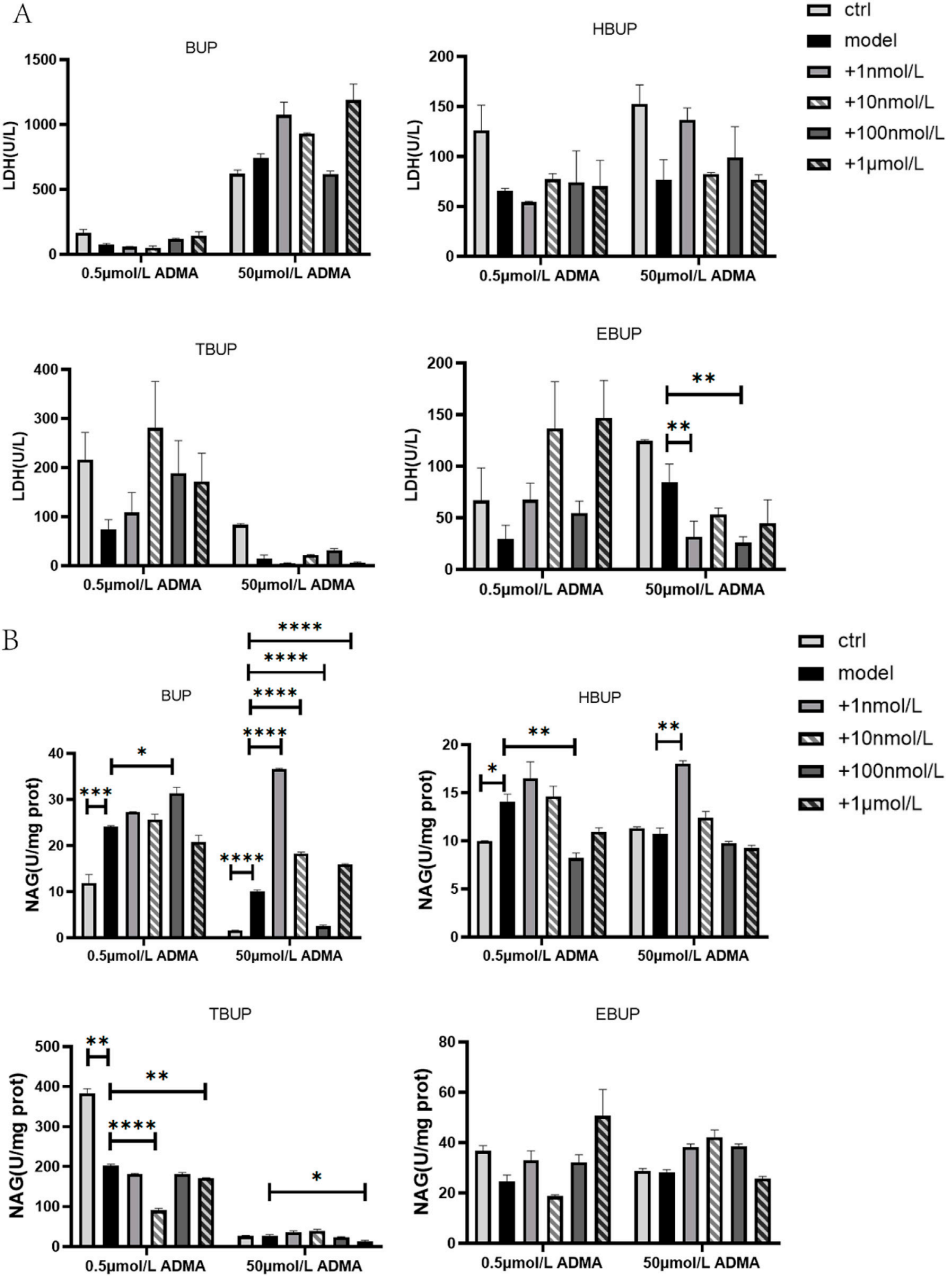
The effect of BUP and BUP metabolites on LDH **(A)** and NAG **(B)** in the culture medium and cell lysate. Ctrl means normal cell group. Data are expressed as mean ± SD (n = 3). *P < 0.05, **P < 0.01, ***P < 0.001, ****P < 0.0001. Data were analyzed by one-way ANOVA.

In the presence of 0.5 μmol/L ADMA, BUP, BUP metabolites and BUP cocktail had no effect on LDH level in cell culture supernatant (Figure 3A). 100 nmol/L HBUP, 10 nmol/L and1 μmol/L TBUP significantly reduced NAG level by 41.61%, 55.45% and 15.74%, respectively (Figure 3B). However, 100 nmol/L BUP significantly increased NAG by 30.33% (Figure 3B).

In the presence of 50 μmol/L ADMA, 1 nmol/L and 100 nmol/L of EBUP significantly reduced LDH level by 62.63% and 69.36%, respectively (Figure 3A). 100 nmol/L BUP and1 μmol/L TBUP significantly reduced NAG by 74.75 % and 52.20%, respectively (Figure 3B).

**FIGURE 4:**
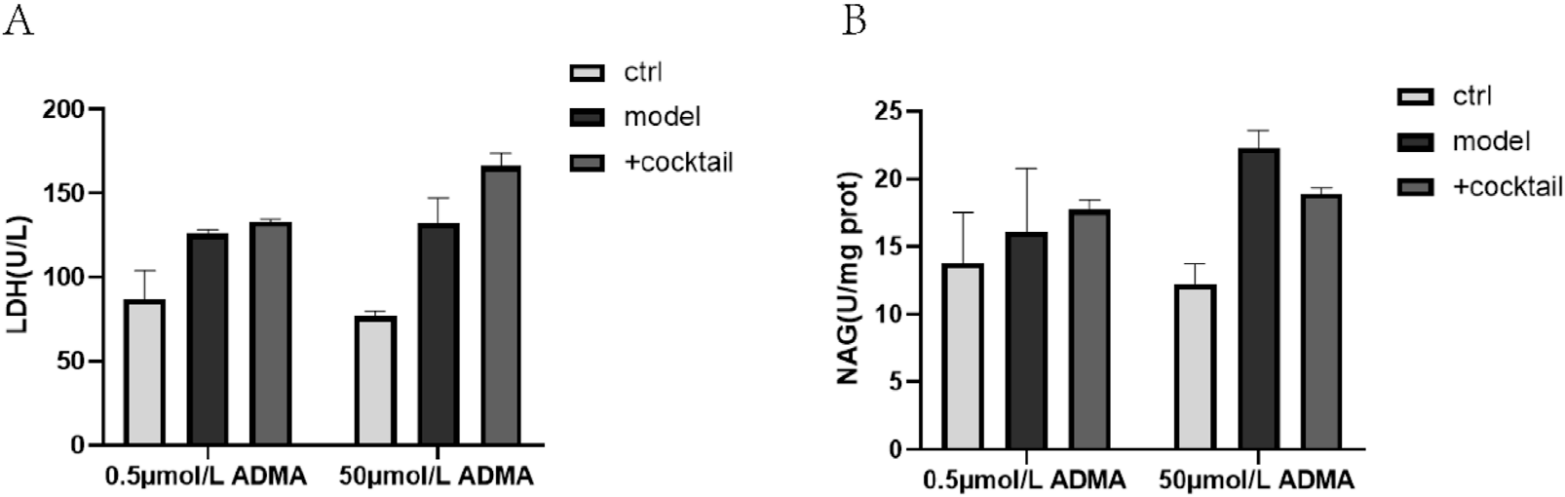
The effect of BUP cocktail on LDH **(A)** and NAG **(B)** in the culture medium and cell lysate. Ctrl means normal cell group. BUP cocktail consisted of 15 nmol/L BUP+125 nmol/L HBUP+50 nmol/L EBUP+50 nmol/L TBUP. Data are expressed as mean ± SD (n = 3). Data were analyzed by one-way ANOVA.

BUP cocktail had no effect on LDH and NAG level in the presence of 0.5 μmol/L and 50 μmol/L ADMA, respectively (Figure 4).

### Establishment of rat model with adenine-induced chronic renal injury

Successful re-produce of the model is indicated by an increase in Cre levels exceeding 180 μmol/L, Bun levels surpassing 50 mmol/L, and histopathological analysis in the kidney tissue sections (Shabaka et al., 2021; Sharp and Brown, 2024).

After 21 days of gavage with adenine, the serum Cre and Bun significantly elevated by 539.80% and 962.43%, respectively (Supplementary Figure S1A). Histological patterns of the kidney exhibited interstitial lesions and necrosis of the renal tubules, which suggested the chronic renal injury models were replicated. (Supplementary Figure S1B).

### The effect of BUP on the biomarkers of serum and urine in rats with chronic renal injury

**FIGURE 5:**
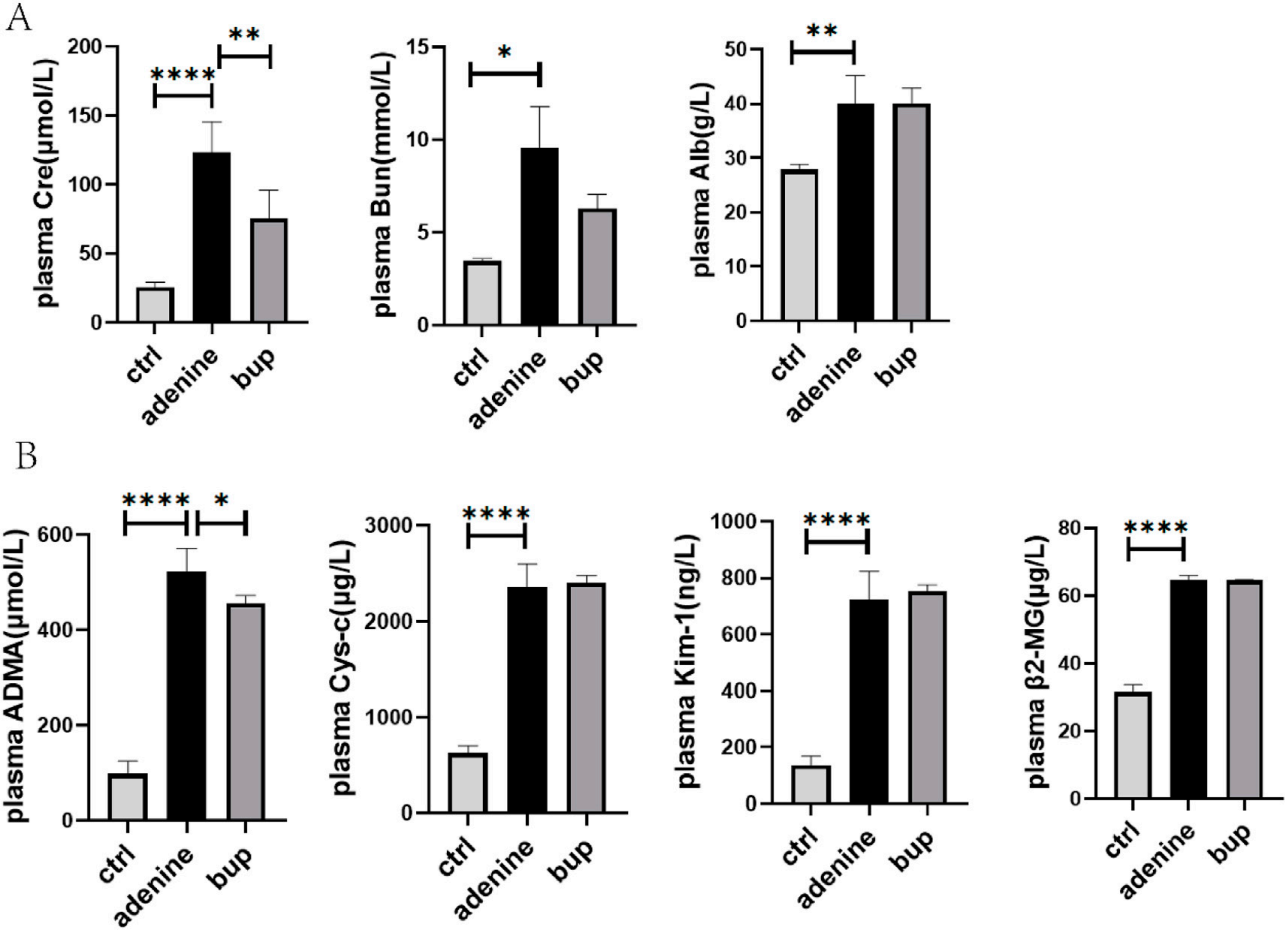
The effects of BUP on Cre, Bun, Alb **(A)** and ADMA, Cys-c, Kim-1, β2-MG **(B)** of plasma in renal injury rats. Data are expressed as mean ± SD (n = 5). *P < 0.05, **P < 0.01, ***P < 0.001, ****P < 0.0001. Data were analyzed by one-way ANOVA.

In rats with chronic renal injury, long-term administration of BUP significantly reduced serum concentration of ADMA and Cre by 12.78% (P = 0.036) and 38.85% (P = 0.019), respectively, but did not significantly affect the plasma concentrations of Cys-c, β2-MG, Kim-1, Bun and Alb (Figures 5A,B).

**FIGURE 6:**
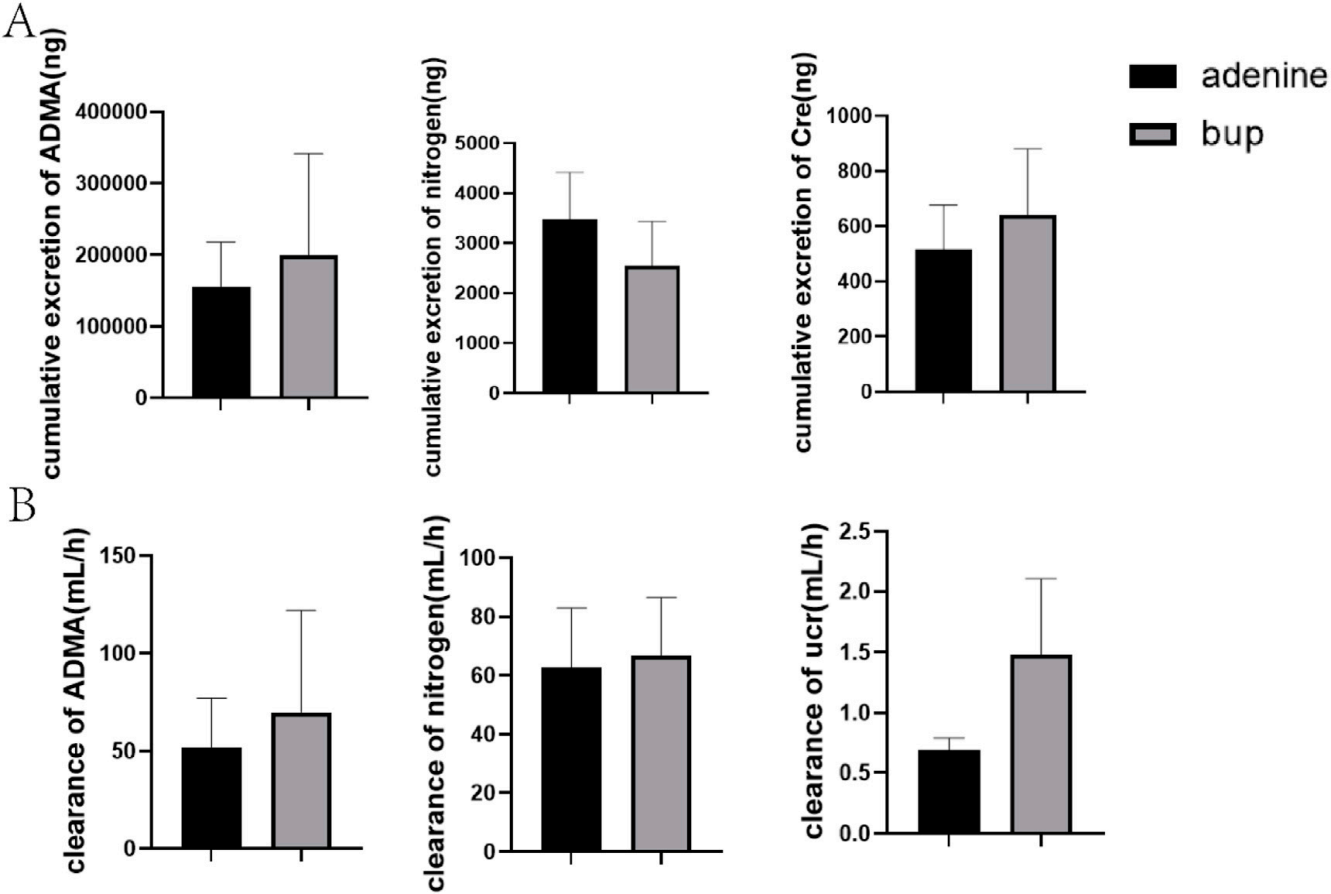
Effects of BUP on urea cumulative excretion **(A)** and urea clearance **(B)** of ADMA, nitrogen and Cre in renal injury rats. Data are expressed as mean ± SD (n = 5). *P < 0.05, **P < 0.01, ***P < 0.001, ****P < 0.0001. Data were analyzed by one-way ANOVA.

Trends of increase in the cumulative amount of ADMA and Cre excreted in urine were observed in the presence of BUP, while they missed statistical significance (Figure 6A). Additionally, the CL_renal_ of ADMA and Cre increased by 35.04% and 114.60%, respectively, but they failed to elicit significant changes (Figure 6B).

### Histopathology

**FIGURE 7:**
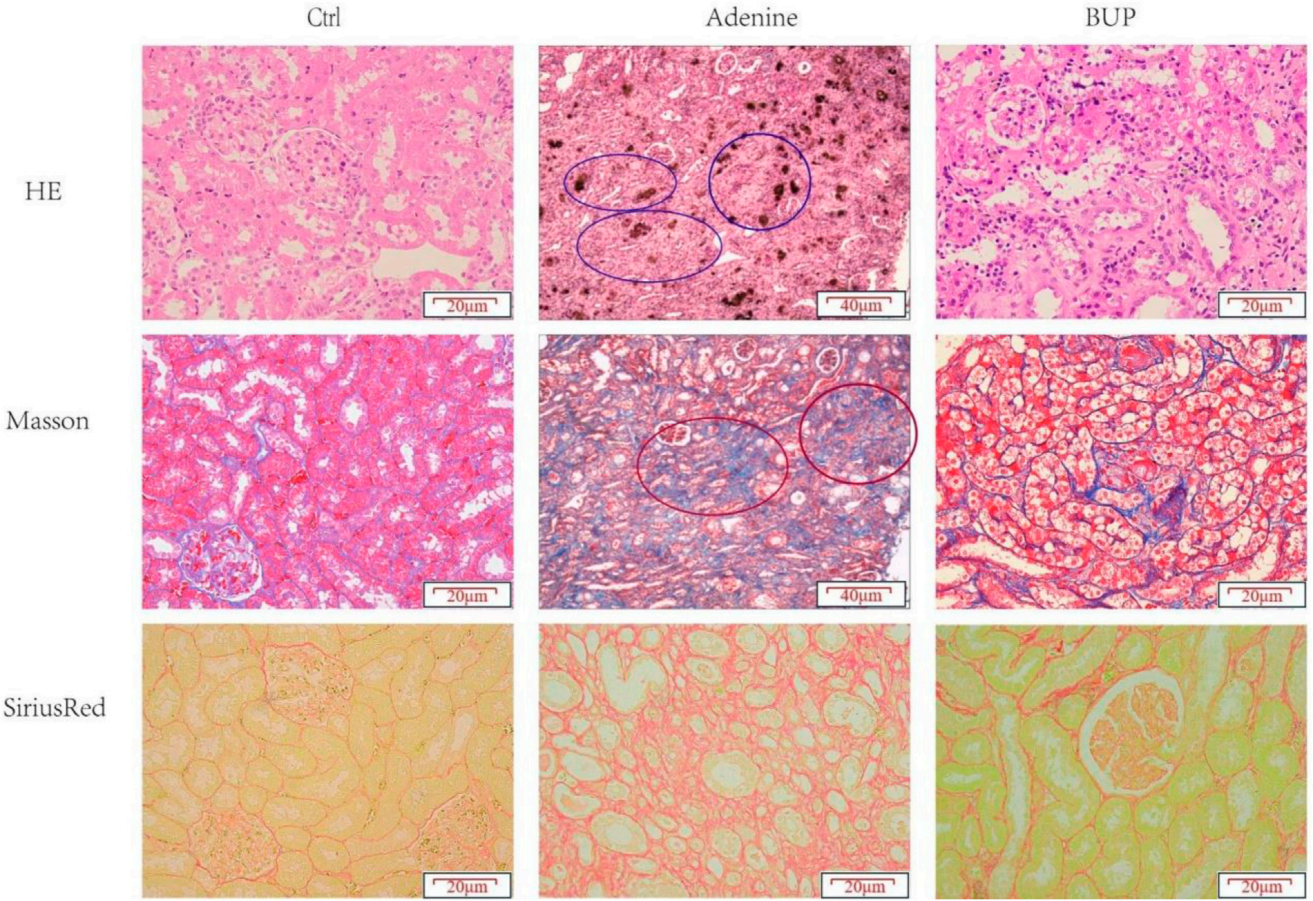
Histopathological evaluation of rat kidney tissue of different groups. Blue circle: tubular atrophy; red circle: interstitial fibrosis.

Compared with the control group, rats with chronic renal injury group presented with tubular atrophy and necrosis, severe interstitial fibrosis, and the confluence of fibrotic areas. After long-term administration of BUP, there was almost no tubular necrosis. Fibrosis was ameliorated, and the tubular basement membrane was slightly thickened (Figure 7).

### The effect of BUP on the expression of Oct2, Ddah1, Oatp4c1 and Mate1

**FIGURE 8:**
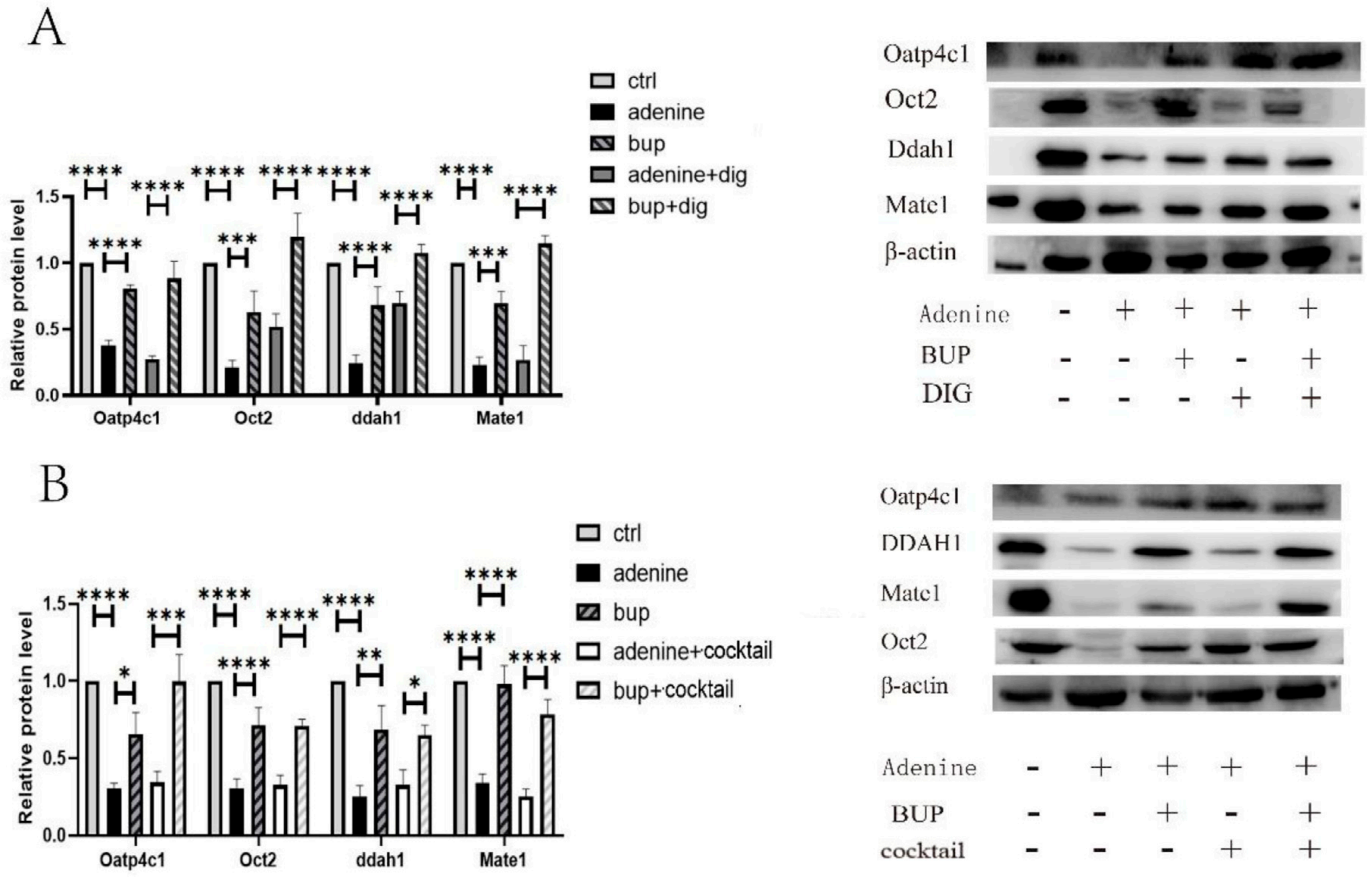
The protein expression of Oatp4c1, Oct2, Ddah1and Mate1 in rat kidney. **(A)** Long-term administration of BUP and followed by single-dose administration of DIG; **(B)** Long-term administration of BUP and followed by single-dose administration of drug cocktail. Cocktail consisted of a single i.v. dose of MET at 5 mg/kg, a single i.v. dose of FUR at 4 mg/kg and a single p.o. dose of RSV at 25 mg/kg. Data are expressed as mean ± SD (n = 5). *P < 0.05, **P < 0.01, ***P < 0.001, ****P < 0.0001. Data were analyzed by one-way ANOVA.

The total protein was extracted from kidney tissue homogenate, and the protein concentrations were determined. The protein expression level of Ddah1, Oatp4c1, Oct2, and Mate1was significantly reduced by 75.50% (*P* < 0.0001), 62.09% (*P* < 0.0001) and 78.79% (*P* < 0.0001), respectively, in chronic renal injury rats (Figure 8). However, long-term administration of BUP significantly increased the protein expression of Ddah1, Oatp4c1, Oct2 and Mate1 by 43.59% (*P* < 0.0001), 42.50% (*P* < 0. 0.0001), 41.94% (*P* < 0. 001) and 46.55% (*P* < 0. 001), respectively (Figure 8).

Additionally, long-term administration of BUP followed by a single dose of DIG resulted in a significant increase in the protein expression of Ddah1, Oatp4c1, Oct2, and Mate1, with upregulation by 37.9% (P < 0.0001), 60.8% (P < 0.0001), 67.65% (P < 0.0001), and 87.6% (P < 0.0001), respectively (Figure 8A). Similarly, after long-term administration of BUP followed by a single dose of MET, there was also a notable increase in the protein expression levels of Ddah1, Oatp4c1, Oct2, and Mate1, which was upregulated by 32.5% (P < 0.05), 65.3% (P < 0.001), 38.14% (P < 0.0001), and 53.07% (P < 0.0001), respectively (Figure 8B).

### Effects of BUP on DIG pharmacokinetics in rats with chronic renal injury

**FIGURE 9:**
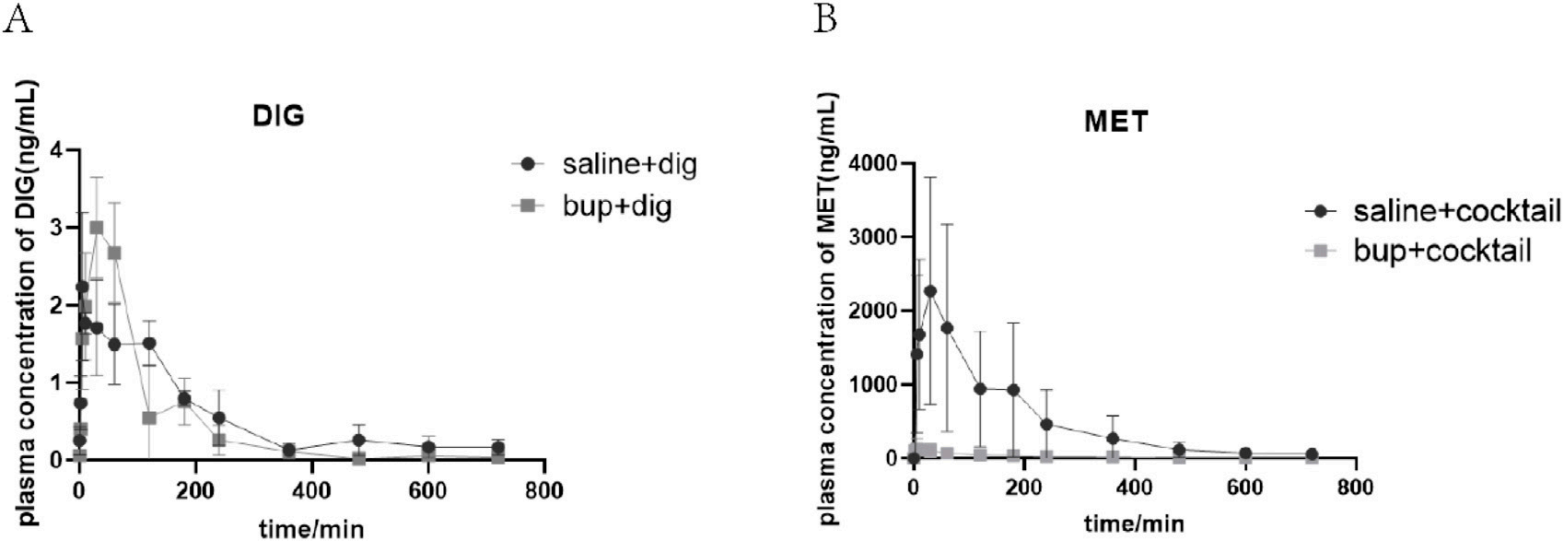
Mean plasma concentration-time profiles of DIG **(A)** and MET **(B)**. Cocktail consisted of a single i.v. dose of MET at 5 mg/kg, a single i.v. dose of FUR at 4 mg/kg and a single p.o. dose of RSV at 25 mg/kg. Data are expressed as mean ± SD (n = 5).

The plasma concentration after intravenous administration of DIG decayed rapidly and biphasically in the presence and absence of BUP (Table 1; Figure 9A). Long-term administration of BUP followed by a single dose of DIG did not significantly affect the systemic plasma concentration, terminal plasma t_1/2_, plasma AUC, C_max_, volume of distribution at steady-state (V_ss_), or total CL of DIG (Table 1; Figure 9A). Although cumulative amount excreted in urine and CL_renal_ of DIG were increased by 82.41% and 70.38%, respectively, they failed to elicit significant changes (Table 1; Figure 9A).

**TABLE 1:** Pharmacokinetic parameters of DIG in rat. (Mean ± SD, n = 5).

Parameters	Adenine + DIG	BUP + DIG
T_1/2_ (h)	3.27 ± 1.14	1.60 ± 0.66
C_max_ (ng/mL)	3.08 ± 1.36	4.31 ± 2.43
AUC_0–12h_ (ng × h/mL)	7.43 ± 4.82	7.88 ± 4.02
AUC_0-∞_ (ng × h/mL)	8.56 ± 5.62	8.12 ± 4.30
CL (mL/h/kg)	1027.71 ± 1032.65	736.92 ± 316.53
Vss (mL/kg)	3,960.33 ± 2,882.68	1629.72 ± 996.86
CL_renal_ (mL/h)	1.17 ± 0.55	1.99 ± 2.13
Amount in Urine (ng)	6.55 ± 3.32	11.94 ± 9.04

Data are expressed as mean ± sd, n = 5. Statistical analysis was performed using one-way ANOVA.

### Effects of BUP on MET pharmacokinetics in rats with chronic renal injury

The plasma concentration after intravenous administration of MET decayed rapidly in the presence of BUP (Table 2; Figure 9B). Long-term administration of BUP followed by a single dose of MET significantly decreased AUC_0–12h_ by 90.35% (*P* = 0.0045) and increased CL_renal_ by 133.52% (*P* = 0.033) (Table 2; Figure 9B). A 615.73% increase in CL, a 2085.67% increase in V_ss_, and a 66.48% decrease in C_max_ were also observed; however, these changes did not reach statistical significance (Table 2; Figure 9B).

**TABLE 2:** Pharmacokinetic parameters of MET in rat. (Mean ± SD,n = 5).

Parameters	Adenine + cocktail	BUP + cocktail
T_1/2_ (h)	3.03 ± 0.86	7.07 ± 5.72
C_max_ (ng/mL)	2,698.87 ± 806.31	904.65 ± 1503.62
AUC_0–12h_ (ng × h/mL)	9050.34 ± 6653.73	873.50 ± 1077.65
AUC_0-∞_ (ng × h/mL)	9786.53 ± 7576.58	1001.68 ± 1056.63
CL (mL/h/kg)	1408.46 ± 768.05	10,080.76 ± 8255.92
Vss (mL/kg)	5353.52 ± 2,688.18	117,010.50 ± 125,153.60
CL_renal_ (mL/h)	4.46 ± 0.92	10.41 ± 4.57
Amount in Urine (ng)	30,542.32 ± 26,845.92	8742.48 ± 10,129.84

Data are expressed as mean ± sd,n = 5. Cocktail consisted of a single i.v. dose of MET, at 5 mg/kg, a single i.v. dose of FUR, at 4 mg/kg and a single p.o. dose of RSV, at 25 mg/kg. Statistical analysis was performed using one-way ANOVA.

### Pharmacokinetics of BUP in rats with chronic renal injury

**FIGURE 10:**
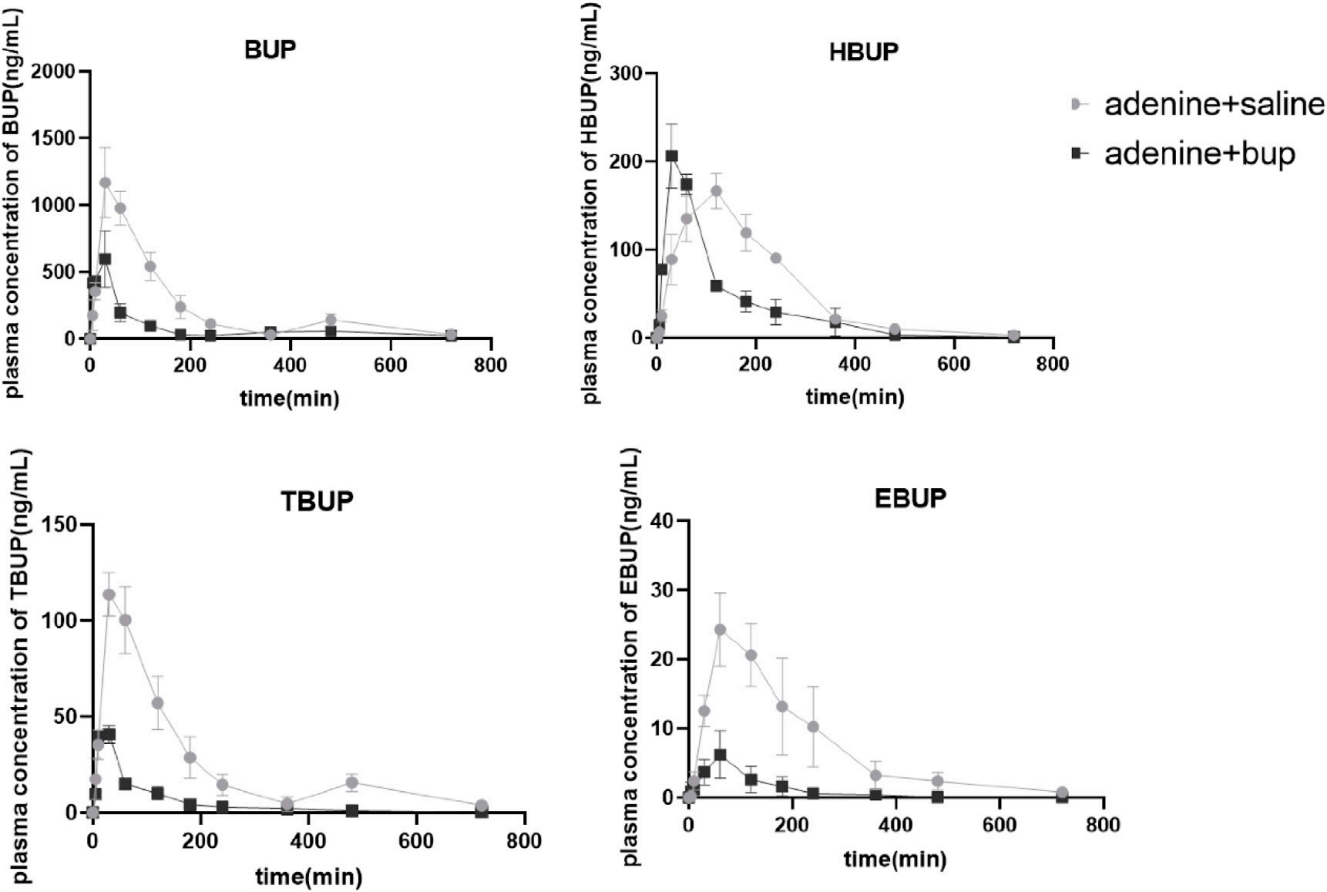
Mean plasma concentration-time profiles of BUP, HBUP, TBUP and EBUP. Data are expressed as mean ± SD (n = 5).

The pharmacokinetic parameters and plasma concentration time profiles of BUP and its circulating metabolites were presented in Table 3 and Figure 10, respectively. Long-term administration of BUP significantly decreased C_max_, AUC_0–12h_, AUC_0-∞_, V_ss_ by 49.50% (*P* = 0.0357), 64.05% (*P* = 0.0025), 59.46% (*P* = 0.0036) and 378.78% (*P* = 0.01), respectively, and significantly increased CL by 146.47% (*P* = 0.0045). Similarly, long-term administration of BUP significantly altered the systematic disposition of its circulating metabolites. The AUC_0–12h_ and AUC_0-∞_ of HBUP was significantly reduced by 33.93% (*P* = 0.0031) and 34.45% (*P* = 0.0051), respectively. AUC_0-∞HBUP_/AUC_0-∞BUP_ was increased by 85.69% (*P* = 0.0213). The C_max_, AUC_0–12h_, AUC_0-∞_ and AUC_0-∞TBUP_/AUC_0-∞BUP_ of TBUP was significantly decreased by 62.79% (*P* = 0.0003), 79.04% (*P* = 0.0010), 80.39% (*P* = 0.0009) and 41.78% (*P* = 0.0019), respectively. The C_max_, AUC_0–12h_, AUC_0-∞_ and AUC_0-∞EBUP_/AUC_0-∞BUP_ of EBUP was significantly decreased by 76.59% (P = 0.0006), 85.55% (P = 0.0053), 85.88% (P = 0.0055) and 59.07% (P = 0.0097), respectively.

**TABLE 3:** Pharmacokinetic parameters of BUP, HBUP, TBUP and EBUP.

Parameters	Saline	bup
	BUP	
T_1/2_ (h)	2.63 ± 0.26	5.11 ± 1.54
C_max_ (ng/mL)	1182.59 ± 248.64	597.22 ± 209.99^*^
AUC_0–12h_ (ng × h/mL)	2,807.57 ± 435.98	1009.37 ± 156.22^**^
AUC_0-∞_ (ng × h/mL)	2,935.00 ± 458.11	1189.68 ± 182.59^**^
CL (mL/h/kg)	13,875.81 ± 2,375.74	34,199.13 ± 5643.67^**^
Vss (mL/kg)	52,339.46 ± 7174.29	250,591.90 ± 74,692.37^*^
	HBUP	
T_1/2_ (h)	2.04 ± 0.82	1.96 ± 0.83
C_max_ (ng/mL)	168.19 ± 19.58	206.06 ± 36.55
AUC_0–12h_ (ng × h/mL)	645.40 ± 57.18	426.40 ± 15.54^**^
AUC_0-∞_ (ng × h/mL)	656.25 ± 68.98	430.19 ± 12.76^**^
AUC_0-∞HBUP_/AUC_0-∞BUP_ (%)	23.24 ± 2.86	43.16 ± 8.95^*^
	TBUP	
T_1/2_ (h)	3.96 ± 2.56	2.02 ± 0.27
C_max_ (ng/mL)	115.63 ± 10.65	43.02 ± 1.55^***^
AUC_0–12h_ (ng × h/mL)	302.76 ± 46.30	63.45 ± 12.21^***^
AUC_0-∞_ (ng × h/mL)	327.40 ± 50.34	64.19 ± 12.68^***^
AUC_0-∞TBUP_/AUC_0-∞BUP_ (%)	10.81 ± 0.80	6.29 ± 0.73^**^
	EBUP	
T_1/2_ (h)	2.55 ± 0.44	1.75 ± 0.46
C_max_ (ng/mL)	26.83 ± 0.87	6.28 ± 3.44^***^
AUC_0–12h_ (ng × h/mL)	88.20 ± 22.88	12.74 ± 6.36^**^
AUC_0-∞_ (ng × h/mL)	90.98 ± 23.94	12.84 ± 6.38^**^
AUC_0-∞EBUP_/AUC_0-∞BUP_ (%)	3.10 ± 0.37	1.27 ± 0.58^**^

Data are expressed as mean ± sd, n = 5, *P < 0.05, **P < 0.01, ***P < 0.001. *: when compared with the saline group. Statistical analysis was performed using one-way ANOVA.

## Discussion

CKD has emerged as a growing global health concern. Depression is considered as one of the most prevalent comorbid psychiatric conditions that occur in patients living with CKD, resulting in a complex interaction that can be detrimental to patient outcomes (Sharp and Brown, 2024). Palmer S et al. reported the prevalence of interview-based depression in people with CKD who require dialysis treatment (CKD stage 5D) was 22.8%, and 21.4% for patients with CKD stages 1–5 in a systematic review (Palmer et al., 2013). Recent studies have shown that 22.0% of patients had depression (stage 4 = 23.8%, stage 5 = 36.8%) in a Moroccan cross-sectional study or even higher as 58.3% in a cross-sectional study at the Jordan University Hospital (Alaoui et al., 2024; Alshelleh et al., 2022). Duan D et al. found that the prevalence of depression was 22.2% in Chinese patients with CKD without dialysis (Duan et al., 2021). Additionally, Chinese cohort study of CKD (C-STRIDE) revealed that the prevalence of depressive symptoms was 37. 8% and increased significantly with CKD stage (Pu et al., 2020). Therefore, it is critical to treat depression in the context of CKD to maximize patient outcomes. However, it remains controversial whether existing pharmacologic interventions are effective for treatment of depression in patients with CKD and end-stage kidney disease (Gregg and Hedayati, 2020). Therefore, there is a growing demand for safer and more innovative antidepressant therapies that not only enhance the effectiveness of depression treatments but also alleviate renal injury in patients with CKD.

BUP, a dopamine-norepinephrine reuptake inhibitor, remains a safe and effective antidepressant and is suitable for first-line use. Dose reduction of bupropion is recommended in patients with CKD3-5 (Nagler et al., 2012). BUP itself undergoes extensive stereoselective metabolism and glucuronidation. Our previous studies have also reported that HBUP, EBUP and TBUP were its major and most abundant circulation metabolites (He et al., 2014; Shen et al., 2018). HBUP, TBUP and EBUP, reach higher plasma concentrations than BUP in human (Costa et al., 2019). After long-term administration of BUP, the steady-state C_max_ and AUC for HBUP and TBUP were 4-, 7-fold, and 4-, 6-fold higher than those following single-dose of BUP, respectively (Costa et al., 2019; Benowitz et al., 2013). HBUP has a longer elimination half-life than BUP (Jefferson et al., 2005). The metabolite potency, relative to BUP, is estimated to be 50%, 20%, and 20% for HBUP, TBUP, and EBUP, respectively. In this context, BUP metabolites are likely to play a very important role in the pharmacological and toxicological effects of the parent drug. The kidney is responsible for 87% bupropion excretion. Our previous studies suggested that the capacity for transporter and enzyme modulation following the administration of BUP in rats and in cynomolgus monkeys (He et al., 2014; Shen et al., 2018). Mao et al. demonstrated that neither BUP nor its active metabolites were substrates for OCT1, OATP1B1, OATP1B3, OATP2B1, OATP4A1, BCRP, MRP2 or P-gp (Han et al., 2019). BUP, HBUP and BUP cocktail (BUP plus its metabolites) significantly stimulated OATP4C1 mediated transport of [^3^H]-DIG, but had no effect on P-gp mediated transepithelial transport of [^3^H]-DIG, (He et al., 2014). However, little is known about their activity at relevant targets (e.g., Ddah1, Oct2, and Mate1). Therefore, BUP and its major circulation metabolites (HBUP, TBUP, EBUP) were chosen.

It is widely recognized that serum levels of asymmetric dimethylarginine (ADMA), a uremic toxin, are significant biomarkers for CKD (Oliva-Damaso et al., 2019; Shafi et al., 2017). Oct2, Mate1, Oatp4c1, and Ddah1 are involved in the renal disposition of ADMA (Strobel et al., 2013). Therefore, it is hypothesized that at clinically relevant plasma concentrations, BUP or its metabolites, modulated Oct2, Oatp4c1 and Mate1 mediated renal transport of ADMA and/or Ddah1 mediated metabolism, which could alleviate the exacerbation of ADMA and retard the progression of renal interstitial lesions and fibrosis.

In healthy serum, the concentration of ADMA is between 0.4 and 0.6 μmol/L (Horowitz and Heresztyn, 2007). In patients with end-stage renal disease, serum concentrations of ADMA increased by 4–10 times and varied a lot (Oliva-Damaso et al., 2019; Shafi et al., 2017). ADMA concentrations were 6.0 ± 0.5 μmol/L and 7.31 ± 0.70 μmol/L in predialysis hemodialysis-treated patients and in hemodialysis-treated patients with manifest atherosclerotic disease, respectively (Kielstein et al., 1999). The ADMA concentrations can be as high as 238.33 ± 16.19 μmol/L in predialysis hemodialysis-treated renal failure patients in samples provided by department of nephrology (n = 8, data not shown). In human endothelial cells, ADMA (0.1 μmol/L to 100 μmol/L) were used to test the inhibition effect on NO formation (Böger et al., 2000). Previous study suggested that intracellular ADMA levels were about 10-fold higher than the reported range for plasma values (Böger et al., 2000). Therefore, a range of ADMA concentrations (0.5 μmol/L, 5 μmol/L, 50 μmol/L) were selected *in vitro* experiments. The presence of 5 and 50 μmol/L ADMA is to mimic pathological conditions. We found ADMA suppressed cell proliferation and promoted cellular apoptosis in HK2 cells in a concentration-dependent manner. Interestingly, under physiological concentration of 0.5 μmol/L ADMA, BUP, BUP metabolites and BUP cocktail had no effect on cellular proliferation and apoptosis. In healthy subjects and adolescents, the concentration range of BUP, HBUP, EBUP plus TBUP are around 0.02–0.6 μmol/L, 0.1–1.68 μmol/L, 0.07–0.56 μmol/L, respectively (Chen et al., 2019; Tsikas, 2020). In smoking haemodialysis patients, the C_max_ of BUP, HBUP, TBUP are 0.38–0.67μM, 1.07–3.04 μmol/L,0.28–1.09 μmol/L, respectively (Worrall et al., 2004). A significant accumulation of BUP metabolites can be observed in smokers with end stage renal disease. The fraction of BUP, HBUP and TBUP unbound in healthy human plasma is ∼0.16, 0.23, 0.58, respectively (Oliva-Damaso et al., 2019; Shafi et al., 2017). Assuming the same unbound fraction, the clinically relevant unbound C_max_ of BUP, HBUP, TBUP in smoking haemodialysis patients are estimated to be 60.8 nmol/L −0.11 μmol/L, 0.25–0.70 μmol/L, 0.16–0.63 μmol/L, respectively. As was hypothesized, BUP and its major circulating metabolite stimulated cell survival in the clinically relevant plasma concentration range (1 nmol/L −1 μmol/L for BUP and 1 nmol/L −100 nmol/L for HBUP) in the presence of 5 μmol/L ADMA. However, only BUP metabolites stimulated cell survival in the clinically relevant plasma concentration range (HBUP at 10 nmol/L and 1 μmol/L for HBUP, 10 nmol/L and 100 nmol/L for TBUP and EBUP, respectively) in the presence of 50 μmol/L ADMA. Moreover, at clinically relevant concentrations, BUP cocktail, consisted of 15 nmol/L BUP, 125 nmol/L HBUP, 50 nmol/L EBUP and 50 nmol/L TBUP, stimulated cell survival in the presence of 5 and 50 μmol/L ADMA. This stimulation modestly reversed the inhibition caused at 5 and 50 μmol/L of ADMA.

NAG, a specific marker for tubular injury and possibly function, were measured (Bosomworth et al., 1999). LDH is an indicator of cellular damage. At physiological concentration, BUP, BUP metabolites and BUP cocktail did not affect cellular injury, as accessed by release of LDH in the culture medium. 100 nmol/L HBUP, 10 nmol/L and1 μmol/L TBUP exhibited protective effects. However, the increase in NAG in the presence of 100 nmol/L BUP was a surprise. At 50 μmol/L ADMA, BUP metabolites, especially 1 nmol/L and 100 nmol/L of EBUP and 1 μmol/L TBUP attenuated cellular damage. It appears that BUP and its metabolites may exert “pleiotropic” effects, with their efficacy in mitigating ADMA-related damage to HK2 cells being contingent upon the exposure of ADMA as well as the concentrations of BUP and its active metabolites.

To further elucidate the mechanisms of protective effect of BUP and its active metabolites in detail, we asked whether a pre-clinical animal model, such as the chronic renal injury rat, could replicate the observation *in vitro*. The adenine-induced CKD model is a well-established model, and it provides certain advantages and limitations (Han et al., 2019; Diwan et al., 2018). Firstly, daily gavage of adenine is simple and rat-friendly. Secondly, this intervention mimics most of the structural and functional changes seen in human CKD, such as renal crystallization and tubulointerstitial fibrosis (HAN et al., 2019; Diwan et al., 2018). Adenine increased serum Bun and Cre concentrations, caused proteinuria, and induced kidney atrophy and fibrosis (Diwan et al., 2018). Thirdly, it is capable of effectively simulating the progression of CKD (mild, moderate and severe) with a high success rate (Han et al., 2019; Diwan et al., 2018). And it can be used to test therapeutic interventions for CKD (Han et al., 2019; Diwan et al., 2018). However, it is inadequate for acute kidney injury -CKD transition research, and it impacts other organs (Han et al., 2019; Diwan et al., 2018). There is a lack of specific biomarkers to identify this model (Han et al., 2019). And the mechanisms of adenine-induced nephron injury remain unclear (Han et al., 2019).

The clinical dose of BUP can be as high as 450 mg/day (Costa et al., 2019). The dosage of 40 mg/kg BUP used in the rat study was human equivalent dose, which was calculated by a human-to-rat equivalent dose ratio of 0.018 based on body surface area, assuming the body weight of 0.2 kg and 70 kg for the rat and human, respectively (Xu et al., 1982). In addition, literature reports indicate that BUP doses of 25, 30, 40, 75, 80 and 160 mg/kg have been administered to rats (Íbias and Nazarian, 2020; Rashidian et al., 2020a; Hall et al., 2015). Therefore, 40 mg/kg of BUP was selected *in vivo*.

Western blot was used to measure the expression of metabolism enzyme and transporter involved in ADMA renal disposition. Consistent with previous reports, the protein expression level of Ddah1, Oatp4c1, Oct2, and Mate1 was significantly reduced in rats with chronic renal injury (Masereeuw et al., 2014; Schwenk and PAI, 2016; Naud et al., 2011). As expected, long-term administration of BUP significantly increased the protein expression of Oatp4C1, Oct2 and Mate1 and Ddah1. ADMA, a uremic toxin, is a substrate for both the basolateral Oct2 and the apical Mate1 in renal tubular epithelial cells (Strobel et al., 2013). Oatp4c1, expressed on the apical membrane of proximal tubule cells, facilitates ADMA reabsorption. Cre is a common biomarker of renal function and is actively secreted across tubular epithelial cells via OCT2 and MATE1/2-K (MA et al., 2023; Nakada et al., 2023). In this study, we found long-term administration of BUP significantly reduced serum concentration of ADMA and Cre by 12.78% and 38.85%, respectively. Cumulative amount of ADMA and Cre excreted in urine and CL_renal_ of ADMA and Cre increased by 28.98% 24.08%,35.04% and 114.60%, respectively, but they failed to reach statistical significance. Large inter-individual variabilities could be one explanation. The significant increase in the expression of Oct2 and Mate 1 would facilitate the secretion of ADMA and Cre across tubular epithelial cells. However, the increased protein expression of Oatp4c1 in rats, namely, stimulation reabsorption, would attenuate the excretion of ADMA in the urine. And the increased Ddah1 expression would also promote the metabolism of ADMA in the tubular epithelial cells. Another possible explanation is that the renal secretion clearance of Cre may not be the major fraction of Cre clearance (CL_renal_ = CL_filtration_ + CL_secretion_-CL_reabsorption_).

The activity of transporter was further evaluated by DDI studies using probe drugs. We selected DIG (Oatp4c1) and MET (Oct2, Mate1). However, due to the broad substrate overlap, it is extremely difficult to select a specific substrate for a certain transporter *in vivo*. Therefore, highly specific *in-vitro* substrates DIG and MET were chosen. This evaluates the net activity of involved transporters, which might’ under/over-estimate the effect of each transporter. DIG is a substrate for P-gp and a substrate for OATP4C1 expressed in the kidney (HE et al., 2014). It is eliminated from the body primarily by renal excretion (70%–85%) and some by non-CYP450 mediated hepatic metabolism (He et al., 2014). It is not a substrate for organic anion-transporting polypeptide transporters OATP1A2, OATP1B1, OATP1B3, and OATP2B1 (Taub et al., 2011). Our previous studies have reported DIG disposition in normal rats and cynomolgus monkeys (He et al., 2014; Shen et al., 2018). And we also have evaluated [3H]-DIG transport by rat-Oatp4c1 and human-OT4P4C1 overexpressing MDCKII cells in the presence and absence of DIG (He et al., 2014). MET has been the recommended probe substrate for OCT2 and MATE1 *in-vitro* by FDA (U.S. Department of Health and Human Services Food and Drug Administration Center for Drug Evaluation and Research CDER, 2020). Cocktail consisting of DIG, MET, FUR and RSV exhibits no mutual pharmacokinetic interactions and have been used for the *in vivo* screening of transporter-mediated DDI in 24 healthy male volunteers (Stopfer et al., 2016; Stopfer et al., 2018).

Results indicated that an 82.41% and 70.38% increase in cumulative amount excreted in urine and CL_reral_ of DIG was observed after chronic administration of BUP, respectively, but they failed to reach statistical significance. It seemed that the long-term administration of BUP may not have significant impact on the activity of Oatp4c1. However, a significant increase in CL_renal_ of DIG has been observed in rat and in cynomolgus Monkey (He et al., 2014; Shen et al., 2018). This inconsistency may be due to a small sample size and large variabilities. Oatp4c1 may possess multiple sites exhibiting highly selective substrate specificity, which differs between ADMA and DIG. And the pharmacokinetic process of DIG is different under pathological conditions of chronic renal injury. Our previous study also reported that the chronic treatment of BUP has greater effect on DIG non-renal elimination pathway rather than CL_renal_, which is likely to be more clinically relevant (SHEN et al., 2018). The significantly increased CL_renal_ and decreased AUC of MET indicated that long-term administration of BUP enhances the activities of Oct2 and Mate1 in rats with chronic renal injury. And this is consistent with the increased expression of Oct2 and Mate1 in Western blot analysis.

There may be other potential mechanisms by which BUP and its metabolites might exert renoprotective effects (e.g., anti-inflammatory effects, antioxidant effects). The anti-inflammatory effects of BUP have been inconsistent in the reported literature (Yetkin et al., 2023; Cámara-Lemarroy et al., 2013; Rashidian et al., 2020b; Karimollah et al., 2021). On the one hand, some studies found that BUP had anti-inflammatory activity through immunomodulation of the macrophages and could reduce the inflammatory response and exert anti-inflammatory influence through suppressing the TLR4 and NF-ĸB expression (Yetkin et al., 2023; Cámara-Lemarroy et al., 2013; Rashidian et al., 2020b). In contrast, other findings implied that BUP had pro-inflammatory effects and should be co-administered with anti-inflammatory medications (Karimollah et al., 2021). There is a lack of direct ‘evidence of antioxidant effects of BUP, but it showed the potential to reduce oxidative stress (Hamdy et al., 2018; Taheri et al., 2018). Additionally, anti-inflammatory effects and antioxidant effects of BUP metabolites remain unclear and should be tested in future. An in-depth study of other potential mechanisms could contribute to the personalized clinical application of BUP in future.

## Conclusion

**FIGURE 11:**
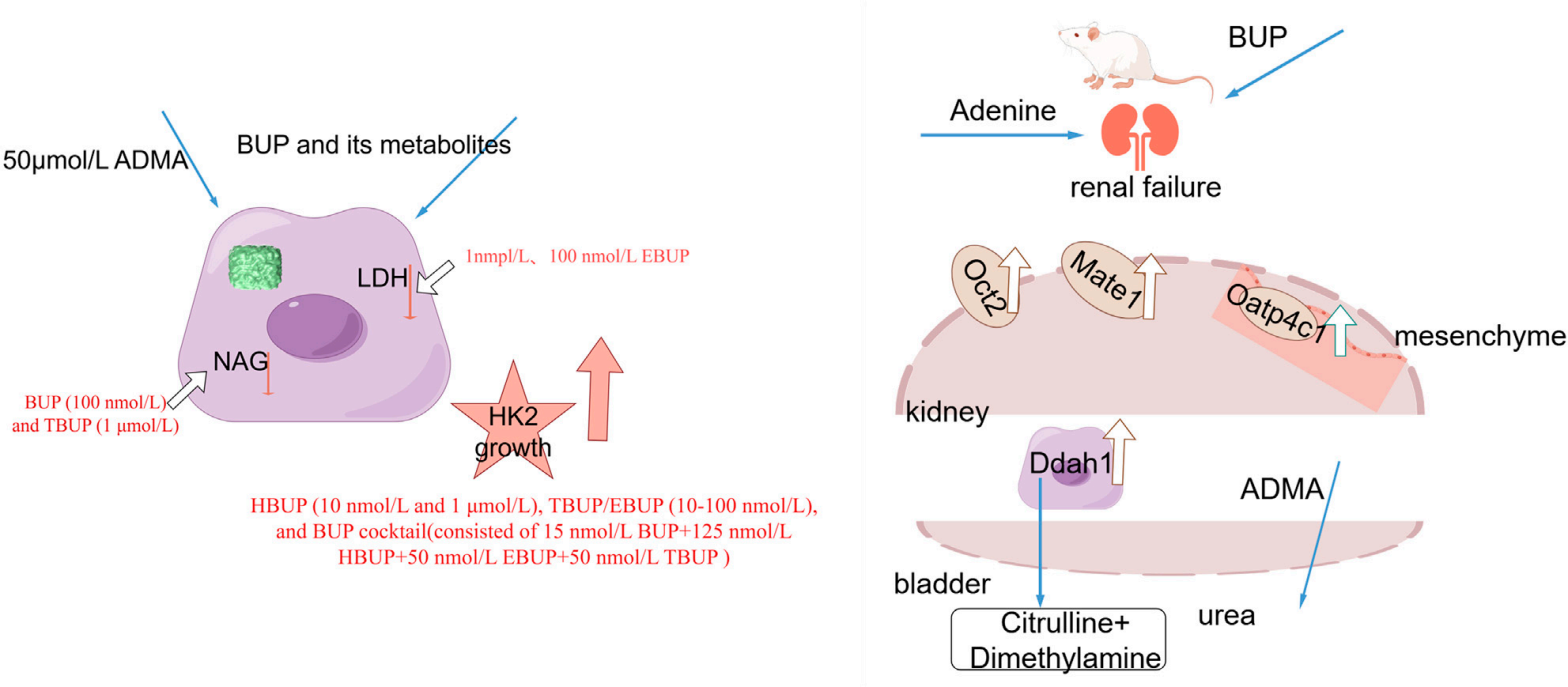
Proposed mechanism (By Figdraw 2.0). At 50 μmol/L ADMA, HBUP (10 nmol/L and 1 μmol/L), TBUP/EBUP (10–100 nmol/L), and BUP cocktail stimulated survival in HK2 cells. EBUP (1/100 nmol/L) lowered LDH. BUP (100 nmol/L) and TBUP (1 μmol/L) decreased NAG. Long term administration of BUP and its metabolites moderately decrease the serum level of ADMA by modulating the Oct2, Mate1, and Oatp4c1-mediated renal transport of ADMA as well as Ddah1-mediated metabolism, which alleviates the exacerbation of ADMA and retards the progression of renal interstitial lesions and fibrosis. ADMA: Asymmetrical Dimethylarginine. BUP: Bupropion. Ddah1: Dimethylarginine dimethylaminohydrolase1. LDH: Lactate dehydrogenase. Mate1: Mammalian multidrug and toxin extrusion protein 1. NAG: N-Acetyl-β-D-glucosidase. Oatp4c1: Organic anion-transporting polypeptide 4c1. Oct2: Organic cation transporter2.

In conclusion, BUP and its metabolites moderately decrease the serum level of ADMA by modulating the Oct2, Mate1, and Oatp4c1-mediated renal transport of ADMA as well as Ddah1-mediated metabolism, which alleviates the exacerbation of ADMA and retards the progression of renal interstitial lesions and fibrosis (Figure 11). To the best of our knowledge, this study represents the first investigation demonstrating that long-term administration of BUP stimulates transporters and metabolic enzymes, leading to reduced plasma exposure of ADMA. Further real-world studies need to be established to ascertain the finding in patients.

## Data Availability

The original contributions presented in the study are included in the article/Supplementary Material, further inquiries can be directed to the corresponding author.

## Glossary

**ADMA**: Asymmetrical Dimethylarginine
**ALB**: Albumin
**AO**: Acridine Orange
**AUC**: the area under the plasma concentration time curve
**Bcrp**: Breast cancer resistance protein
**Bun**: Urea nitrogen
BUP: Bupropion
**CCK-8**: Cell Counting Kit-8
**CKD**: Chronic kidney disease
**C**_**max**_: peak plasma concentration
**Cre**: Creatinine
**CYP**: Cytochrome P450
**Cys-c**: Cystatin-C
**Ddah1**: Dimethylarginine dimethylaminohydrolase1
**DIA**: Diazepam
**DIG**: Digoxin
**DMEM**: Dulbecco’s Modified Eagle Medium
**EB**: Ethidium Bromide
**EBUP**: Erythro-hydrobupropion
**ECL**: Enhanced Chemiluminescence
**ESI**: Electro-Spray Ionization
**FUR**: Furosemide
**HBUP**: Hydroxybupropion
**HK2**: Human kidney 2
**HRP**: Horseradish Peroxidase
**KIM-1**: Kidney injury molecule-1
**LC**: Liquid chromatography
**LDH**: Lactate dehydrogenase
**Mate1**: Mammalian multidrug and toxin extrusion protein 1
**MET**: Metformin
**MRM**: Multiple Reaction Monitoring
**Mrp2**: Multidrug resistance-associated protein 2
**MS/MS**: Tandem mass spectrometry
**NAG**: N-Acetyl-β-D-glucosidase
**CL**_**renal**_: Renal Clearence
**Oatp**: Organic anion-transporting polypeptide
**Oct2**: Organic cation transporter2
**P-gp**: P-glycoprotein
**PMSF**: Phenylmethanesulfonyl fluoride
**PVDF**: Polyvinylidene fluoride
**RSD RSV**: Relative Standard Deviation Rosuvastatin
**SD**: Standard Deviation
**TBUP**: Threo-hydrobupropion
**T**_**1/2**_: Terminal half-life
**UGT**: Uridine Diphosphate Glycosyltransferase
**β2-MG**: β2-microglobulin
