# Supplementary material for: Bupropion decreases plasma levels of asymmetric dimethylarginine and ameliorates renal injury by modulation of Ddah1, Oatp4c1, Oct2, and Mate1 in rats with adenine-induced chronic renal injury
Source: Front Pharmacol. 2025 May 22;16:1565713. doi: 10.3389/fphar.2025.1565713 (PMC12138378; doi:10.3389/fphar.2025.1565713)
Supplement: Supplementary file 1 [file Supplementaryfile1.pdf]

Supplemental materials

Supplemental Tab 1. Pharmacokinetic parameters of RSV in rat. (Mean ± SD，n=5).

Parameters	Adenine + cocktail	BUP + cocktail
$T_{1/2}$ (h)	4.72±0.639	4.34±1.42
C_{max} (ng/mL)	903.48±203.75	626.07±239.02
AUC_{0-12h} (ng×h/mL)	2124.12±1366.96	2784.36±1041.83
$AUC_{0-\infty}$ (ng×h/mL)	3136.59±1437.20	3516.95±1163.02
CL (mL/h/kg)	10321.06±5270.27	7857.95±3081.50
Vss (mL/kg)	62356.96±33507.62	50018.46±27252.10
CL_{renal} (mL/h)	0.054±0.055	0.116±0.034
Amount in Urine (ng)	62.33±50.91	317.49±151.97*

Note: cocktail consisted of a single i.v. dose of MET at 5 mg/kg, a single i.v. dose of FUR at 4 mg/kg and a single p.o. dose of RSV at 25 mg/kg.

Supplemental Tab 2. Pharmacokinetic parameters of FUR in rat. (Mean ± SD，n=5).

Parameters	Adenine + cocktail	BUP + cocktail
$T_{1/2}$ (h)	1.68±0.31	2.07±0.34
C_{max} (ng/mL)	18539.16±40508.07	36056.61±43575.79
AUC_{0-12h} (ng×h/mL)	34021.01±6819.85	38142.70±14336.41
$AUC_{0-\infty}$ (ng×h/mL)	34346.64±7005.39	39103.20±15034.05
CL (mL/h/kg)	169.85±14.82	120.69±65.33
Vss (mL/kg)	383.49±18.90	353.10±174.44
CL_{renal} (mL/h)	0.31±0.10	0.37±0.28
Amount in Urine (ng)	5791.89±6288.91	11972.00±6364.85

Note: cocktail consisted of a single i.v. dose of MET at 5 mg/kg, a single i.v. dose of FUR at 4 mg/kg and a single p.o. dose of RSV at 25 mg/kg.

Supplemental Tab 3. Precision and accuracy of BUP, HBUP, TBUP, EBUP, DIG, MET, RSV and

FUR in rat plasma. (Mean ± SD, n=6)

Analyte	Spiked conc. (ng/mL)	Measured conc. (ng/mL)		
		Mean conc.±S.D. (ng/mL)	RSD (%)	Bias (%)
BUP	0. 25	0.251±0.017	6.73	0.33
	20	20.62 ±0.98	4.75	3.09
	500	531.21±17.76	3.34	6.24
HBUP	0. 25	0.259±0.027	10.0	3.44
	20	19.41 ±0.85	4.40	-2.97
	500	470.74±15.16	3.22	-5.85
TBUP	0.25	0.262±0.018	6.87	4.73
	20	20.80± 0.969	4.66	3.99
	500	517.69±15.07	2.91	3.54
EBUP	0.25	0.247±0.021	8.48	-1.33
	20	20.79±1.02	4.89	3.95
	500	538.35±17.51	3.25	7.67
DIG	0.125	0.13±0.0058	4.42	5.28
	10	9.44 ±0.67	7.12	-5.57
	400	385.19±20.80	5.40	-3.70
MET	0. 25	0.251±0.0075	2.99	0.20
	20	18.54±0.94	5.08	-7.29
	500	525.12±12.23	2.33	5.02
RSV	0. 25	0.255±0.019	0.074	2.00
	20	20.58±0.69	0.034	2.92
	500	467.12±5.90	0.012	-6.58
FUR	0. 25	0.258±0.0078	0.030	3.00
	20	19.61±0.89	0.046	-1.94

	500	452.34±10.20	0.022	-9.53

Supplemental Tab 4. Precision and accuracy of BUP, HBUP, TBUP, EBUP, DIG, MET, RSV and FUR in rat urine. (Mean ± SD，n=6)

Analyte	Spiked conc. (ng/mL)	Measured conc. (ng/mL)		
		Mean conc.±S.D. (ng/mL)	RSD (%)	Bias (%)
BUP	0. 25	0.24±0.023	10.02	-6.53
	20	22.66±0.40	1.76	13.29
	500	485.34±9.17	1.89	-2.93
HBUP	0. 25	0.28±0.0025	0.90	11.4
	20	22.02±0.60	2.74	10.10
	500	479.60±11.58	2.41	-4.08
TBUP	0.25	0.238±0.011	4.99	-4.67
	20	19.01±0.49	2.57	-4.94
	500	486.21±11.69	2.41	-2.76
EBUP	0.25	0.240±0.014	5.61	-3.80
	20	20.73±0.52	2.50	3.64
	500	498.10±12.77	2.56	-0.38
DIG	0.125	0.120±0.0049	4.08	-4.0
	10	10.51±0.475	4.52	5.08
	400	459.487±2.72	0.592	-14.87
MET	0. 25	0.244±0.014	5.89	-2.27
	20	20.68±1.37	6.62	3.42
	500	482.25±23.42	4.86	-3.55
RSV	0. 25	0.232±0.0050	0.021	-7.40
	20	20.14±0.62	0.031	0.72

	500	487.36±2.71	0.0056	-2.53
FUR	0. 25	0.235±0.023	0.096	-6.00
	20	19.74±1.06	0.054	-1.31
	500	528.66±47.71	0.090	5.73

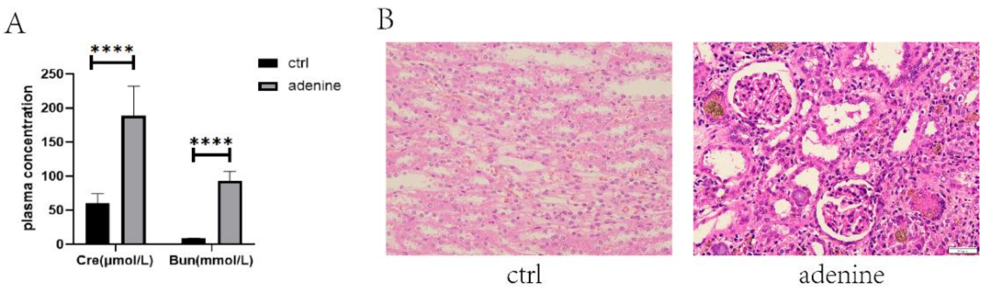

Supplemental Figure 1. The concentrations of plasma Cre and bun (A) and HE staining of kidney tissues in renal failure model of rat. (n=5, Mean ± SD, **P* < 0.05, ***P* < 0.01, ****P* < 0.001, *****P* < 0.0001.)

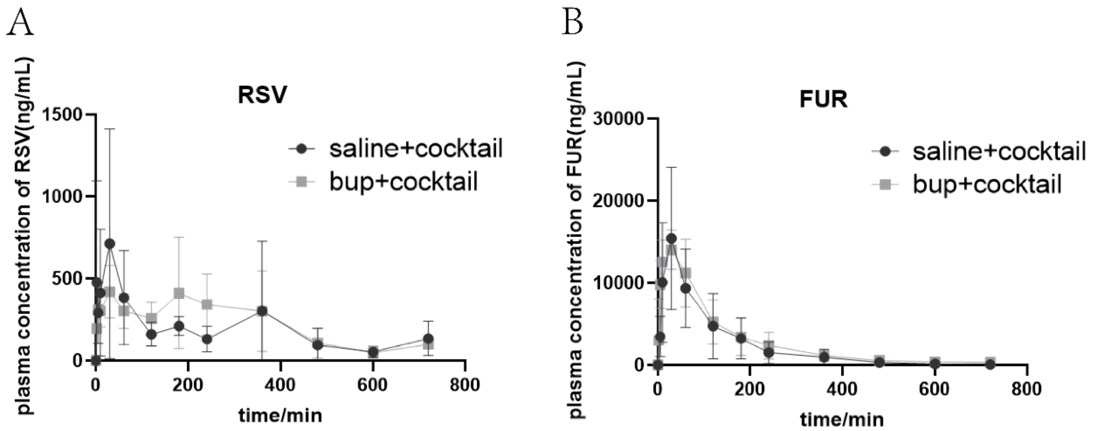

Supplemental Figure 2. Mean plasma concentration-time profiles of RSV and FUR. Data are expressed as Mean ± SD (n = 5). Cocktail consisted of a single i.v. dose of MET at 5 mg/kg, a single i.v. dose of FUR at 4 mg/kg and a single p.o. dose of RSV at 25 mg/kg.

DIA

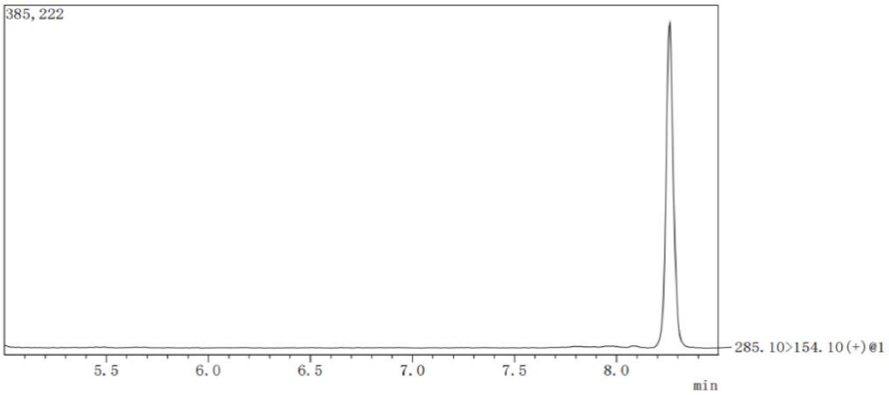

DIG

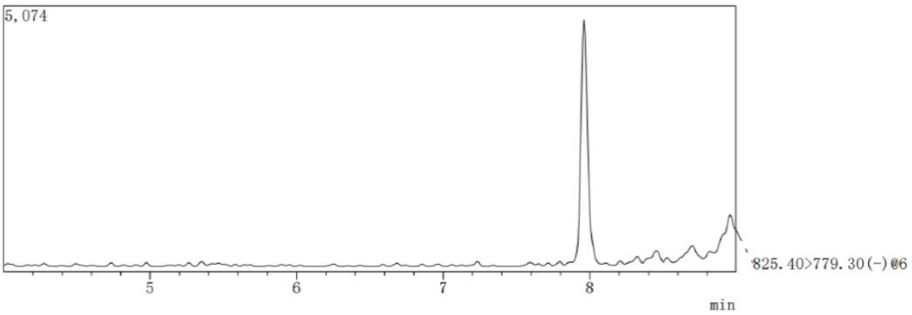

BUP

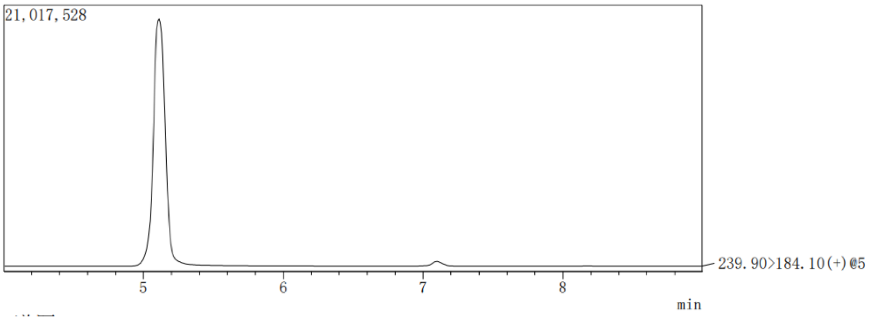

HBUP

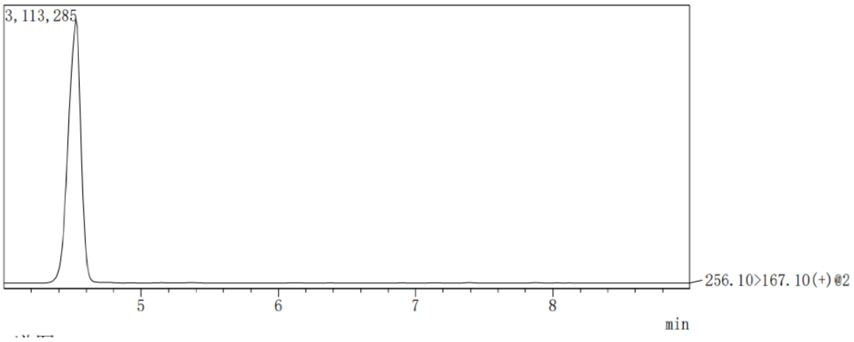

TBUP

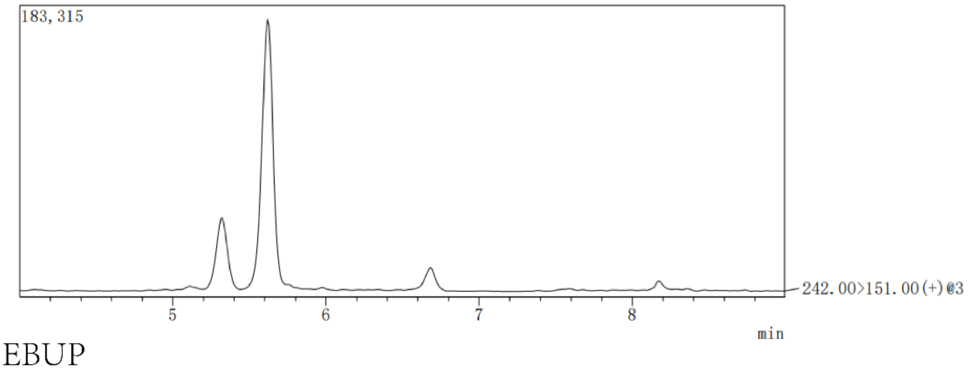

EBUP

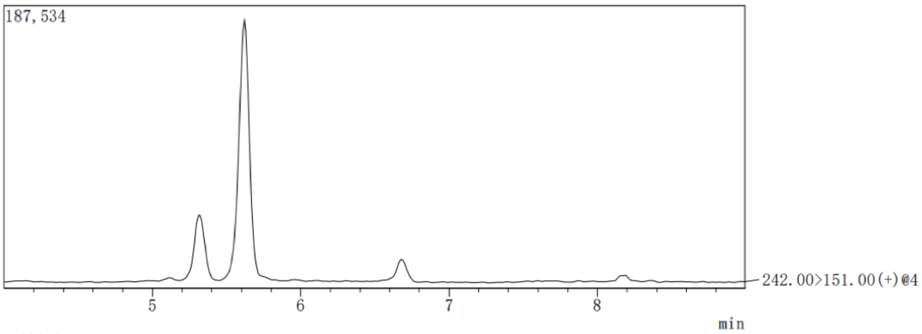

MET

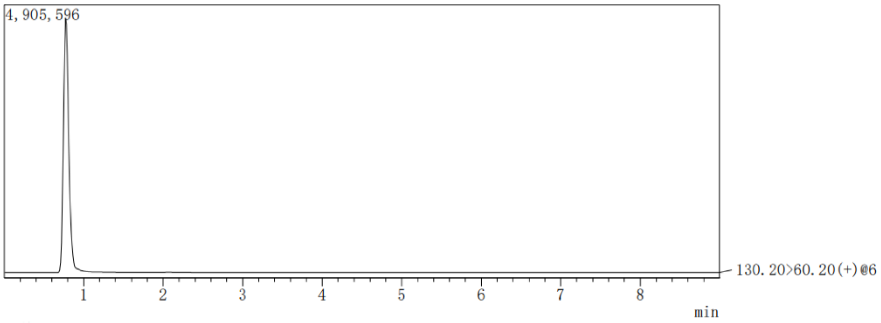

RSV

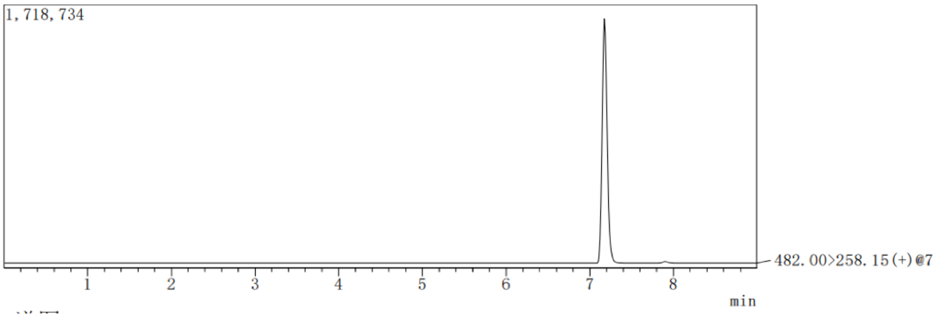

FUR

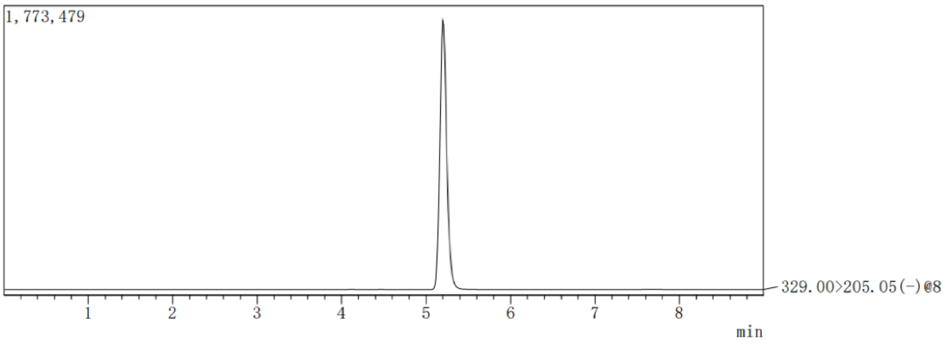

Supplemental Figure 3. Chromatograms of DIA(IS), DIG, BUP, HBUP, TBUP, EBUP, MET, RSV and FUR in rat plasma samples by LC-MS/MS.

DIA

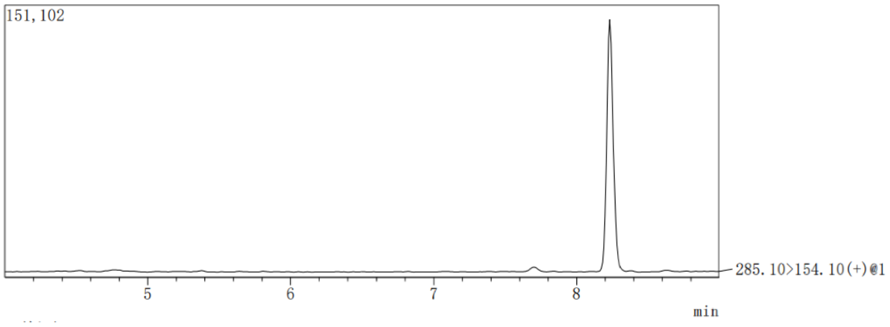

DIG

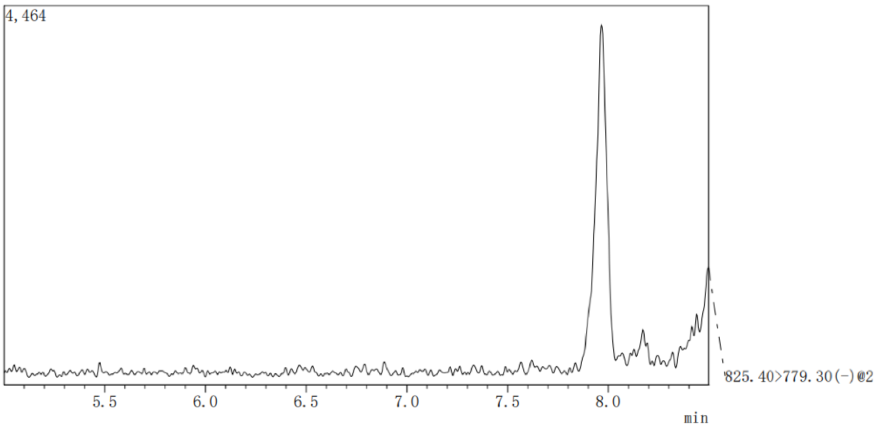

BUP

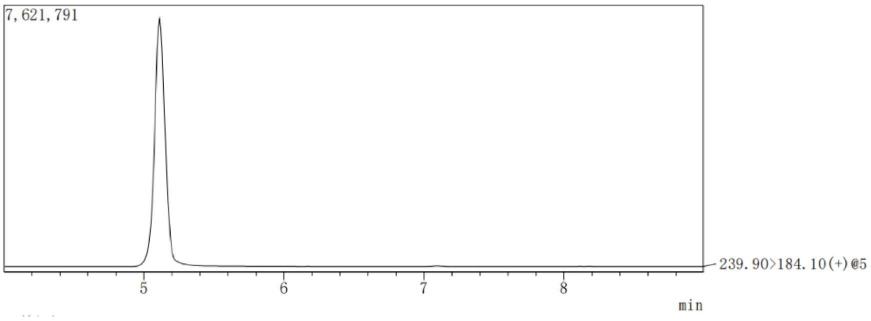

HBUP

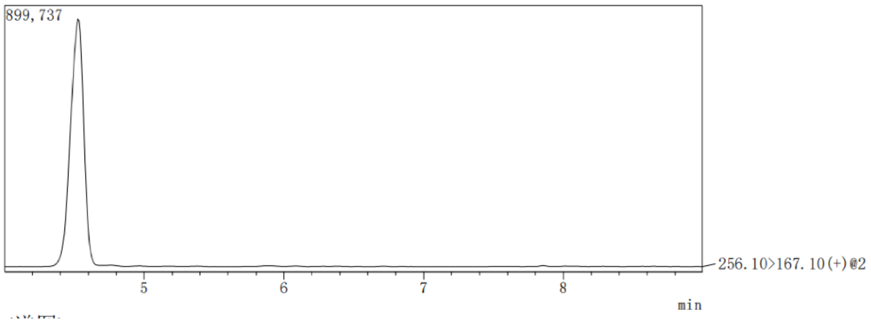

TBUP

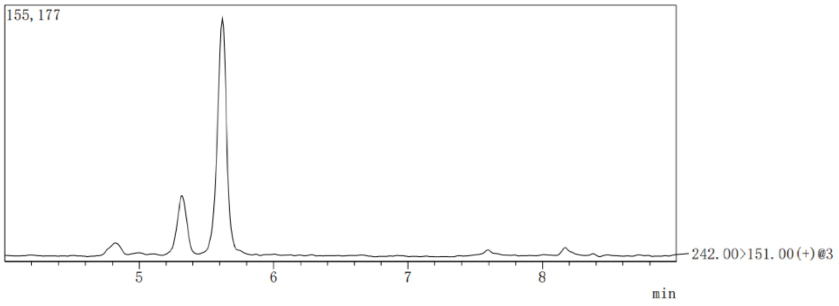

EBUP

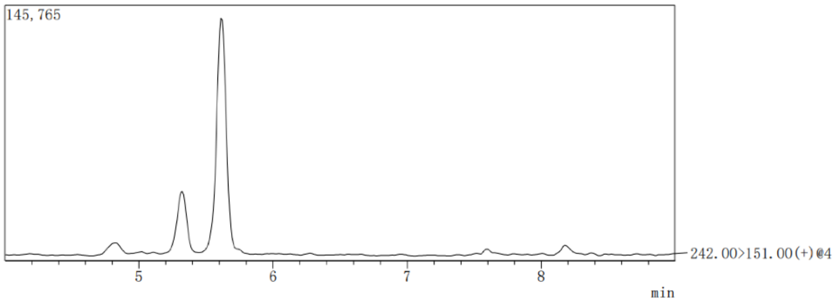

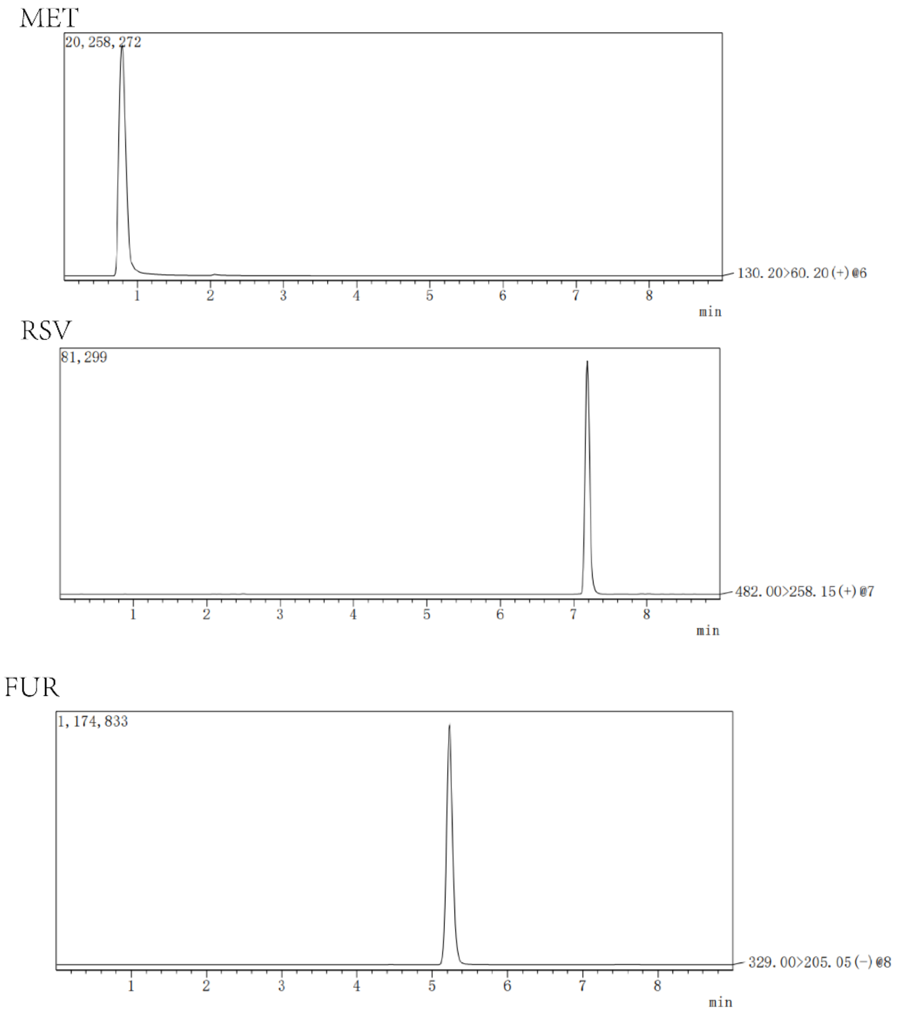

Supplemental Figure 4. Chromatograms of DIA(IS), DIG, BUP, HBUP, TBUP, EBUP, MET, RSV and FUR in rat urine samples by LC-MS/MS.

DIG

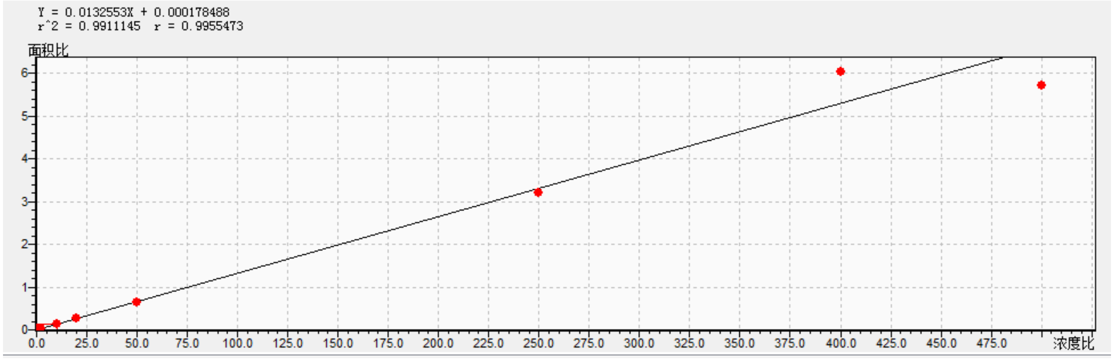

HBUP

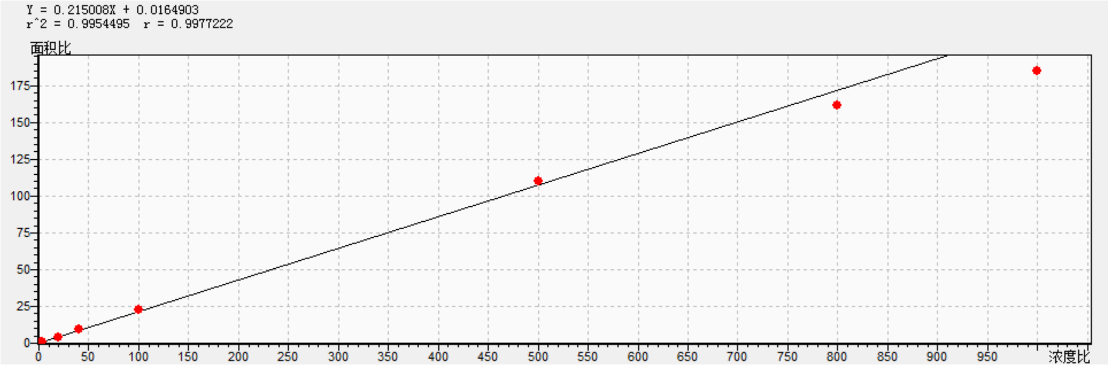

TBUP

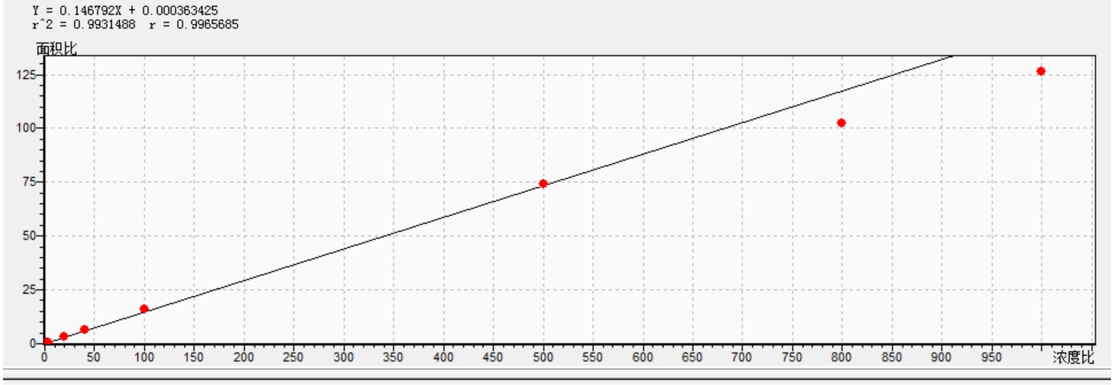

EBUP

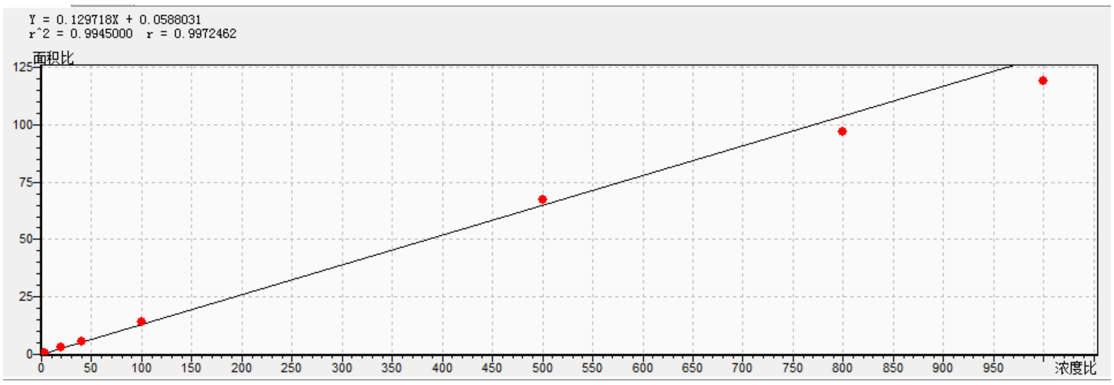

BUP

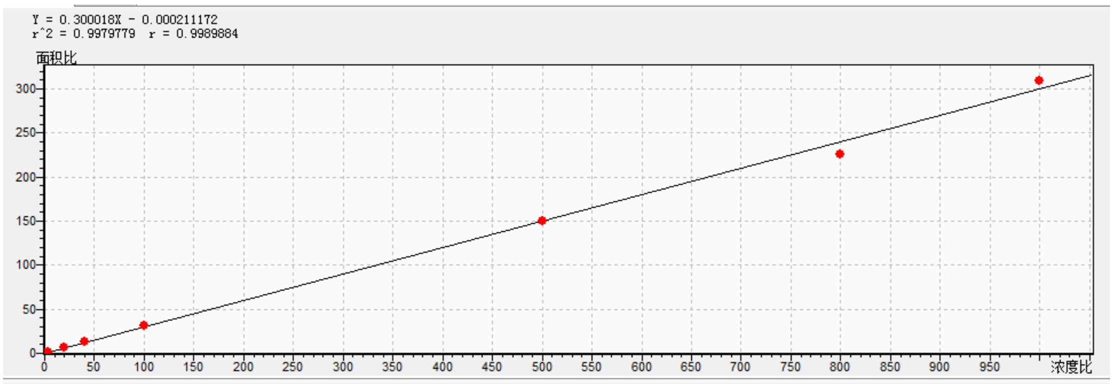

MET

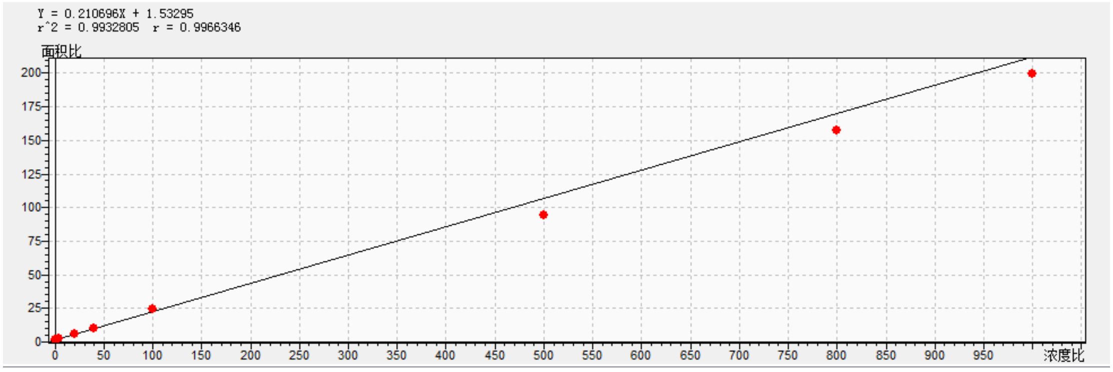

RSV

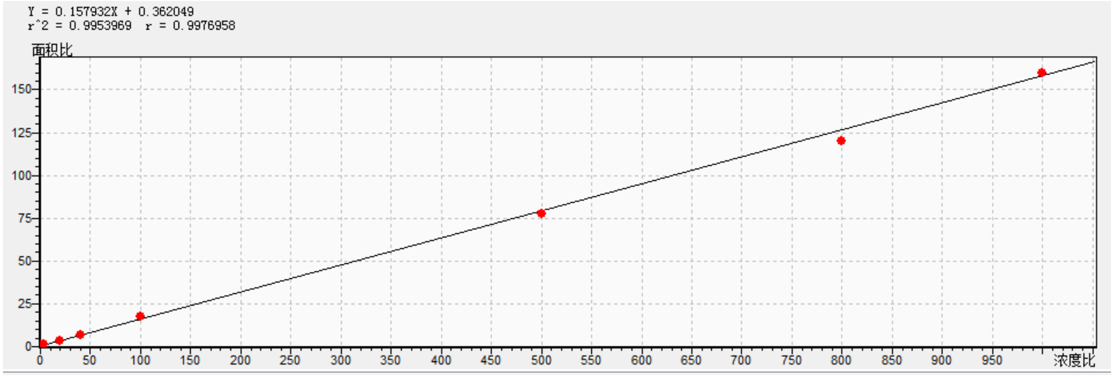

FUR

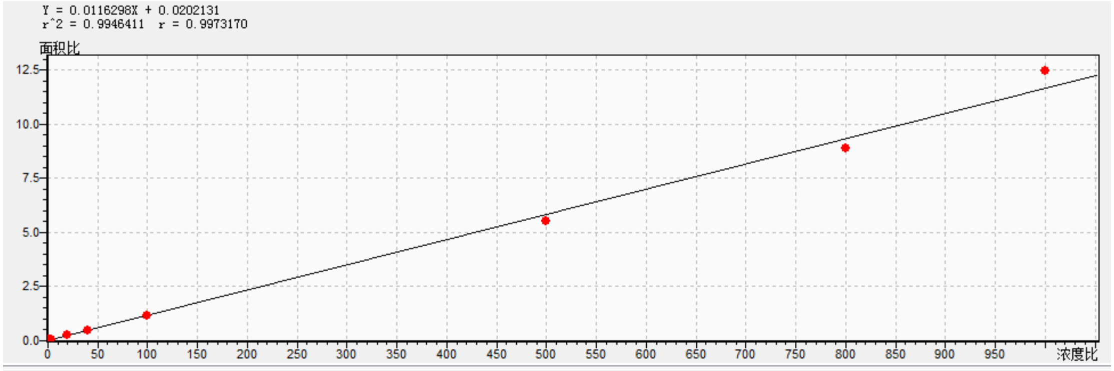

Supplemental Figure 5. Standard curves of DIG, BUP, HBUP, TBUP, EBUP, MET, RSV and FUR in rat plasma.

DIG

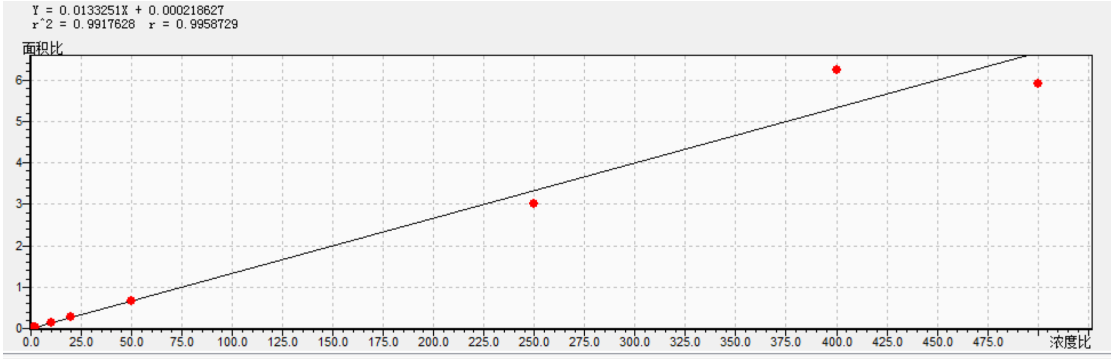

BUP

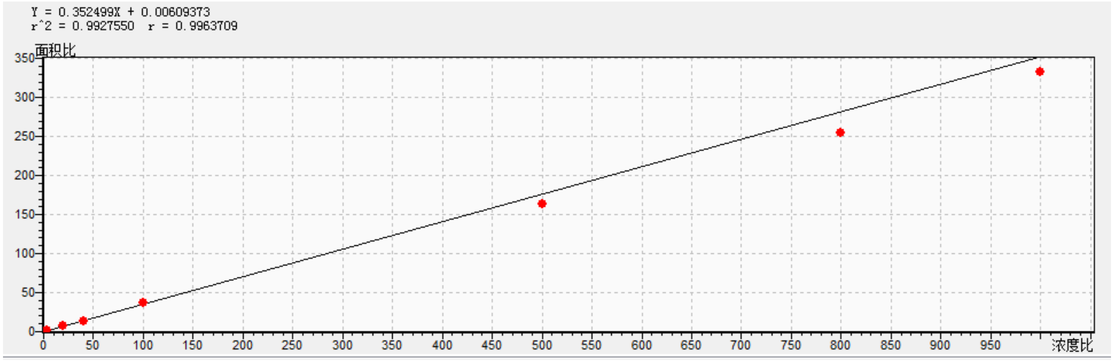

HBUP

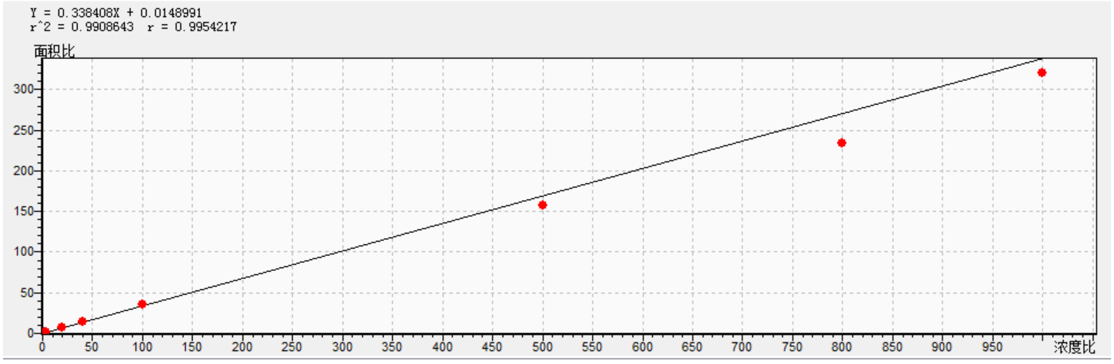

TBUP

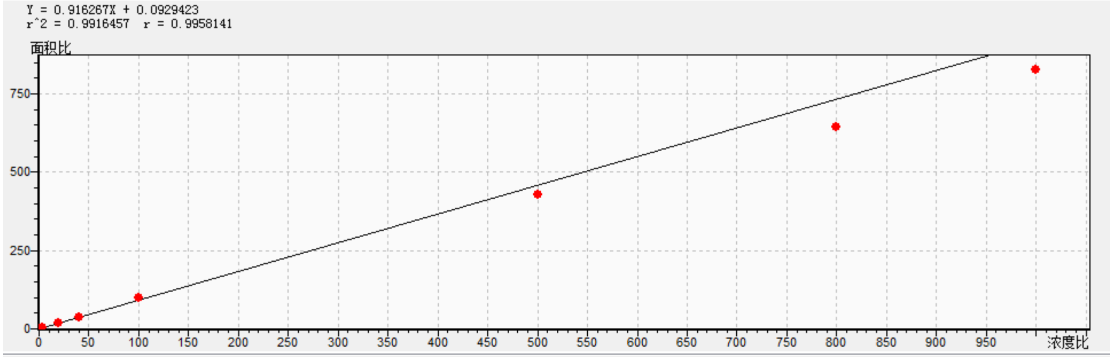

EBUP

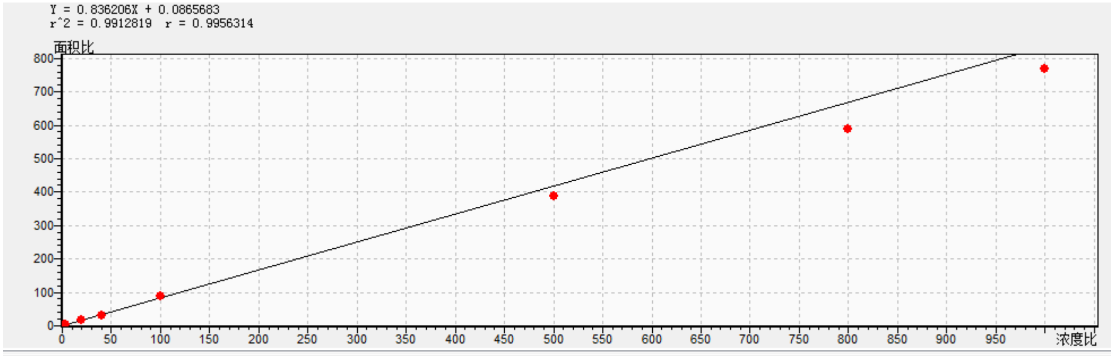

MET

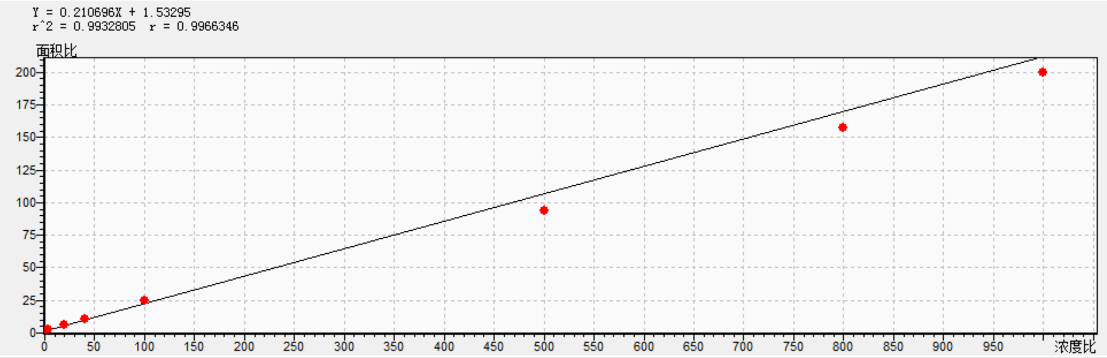

RSV

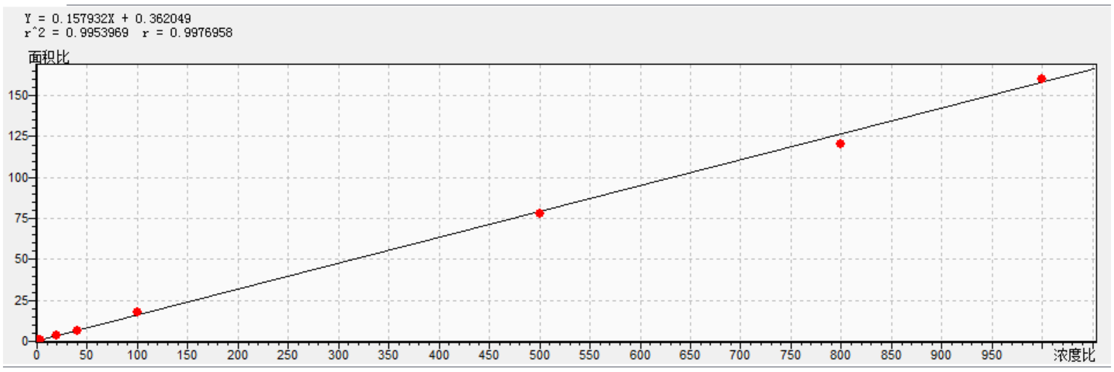

FUR

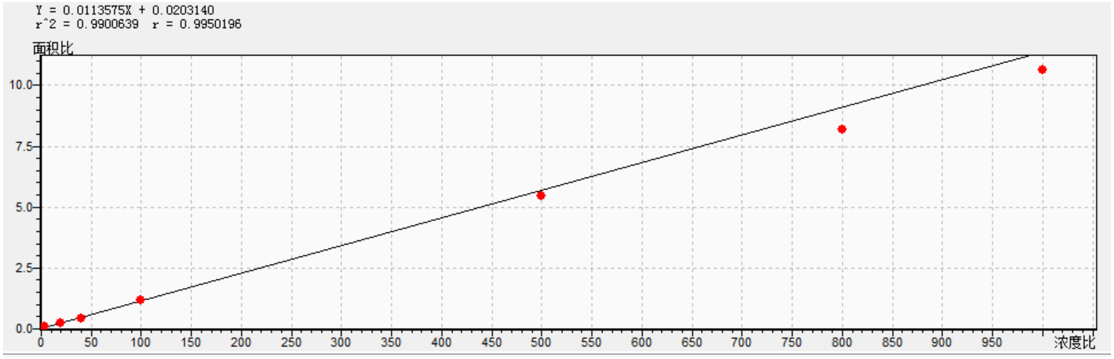

Supplemental Figure 6. Standard curves of DIG, BUP, HBUP, TBUP, EBUP, MET, RSV and FUR in rat urine.
